# An intermolecular FRET sensor detects the dynamics of T cell receptor clustering

**DOI:** 10.1038/ncomms15100

**Published:** 2017-04-28

**Authors:** Yuanqing Ma, Elvis Pandzic, Philip R. Nicovich, Yui Yamamoto, Joanna Kwiatek, Sophie V. Pageon, Aleš Benda, Jérémie Rossy, Katharina Gaus

**Affiliations:** 1 EMBL Australia Node in Single Molecule Science, School of Medical Sciences, University of New South Wales, Sydney, New South Wales 2052, Australia; 2 ARC Centre of Excellence in Advanced Molecular Imaging, University of New South Wales, Sydney, New South Wales 2052, Australia; 3 Biomedical Imaging Facility, Lowy Cancer Research Centre, University of New South Wales, High Street, Kensington, Sydney, New South Wales 2052, Australia

## Abstract

Clustering of the T-cell receptor (TCR) is thought to initiate downstream signalling. However, the detection of protein clustering with high spatial and temporal resolution remains challenging. Here we establish a Förster resonance energy transfer (FRET) sensor, named CliF, which reports intermolecular associations of neighbouring proteins in live cells. A key advantage of the single-chain FRET sensor is that it can be combined with image correlation spectroscopy (ICS), single-particle tracking (SPT) and fluorescence lifetime imaging microscopy (FLIM). We test the sensor with a light-sensitive actuator that induces protein aggregation upon radiation with blue light. When applied to T cells, the sensor reveals that TCR triggering increases the number of dense TCR–CD3 clusters. Further, we find a correlation between cluster movement within the immunological synapse and cluster density. In conclusion, we develop a sensor that allows us to map the dynamics of protein clustering in live T cells.

## Abstract (web summary)

Cellular signalling is often facilitated by membrane protein clustering, but detection of protein clustering at high spatiotemporal resolution is challenging. Here the authors develop a single-chain FRET sensor they name CliF to look at intermolecular associations and dynamics of TCR-CD3 clusters on the T cell surface.

The signalling activity of many membrane proteins depends on their nanoscale clustering into functionally distinct domains^1^,^2^,^3^. For example, ligand-induced T-cell receptor (TCR) clustering has been linked to the initiation of intracellular signalling, leading to T-cell activation and initialization of an immune response^4^. Indeed, most of the components of the TCR signalosome dynamically assemble within microclusters in an actin-dependent manner^5^,^6^,^7^. It is thought that the resulting signalling platforms initiate and amplify TCR signalling. For instance, TCR signalling relies on clustering and co-clustering with the Src-family kinase Lck, which is responsible for the phosphorylation of the TCR–CD3 complex^5^,^8^. Thus, the importance of mapping the spatiotemporal dynamics of protein clustering has become increasingly apparent, especially in the context of membrane signalling.

The technical challenges of measuring protein clusters in live cells are set by two parameters. First, clustering often involves only a small fraction of the expressed proteins. Thus, the technique must be able to detect a few protein clusters amongst a background of non-clustered molecules. Single-molecule localization microscopy has successfully addressed this challenge by imaging individual proteins and employing cluster analyses that detect non-random distributions in point patterns^8^,^9^,^10^. However, extending this imaging technology to live cells has not been trivial^11^. The second challenge is the fast kinetics of protein clustering on the timescale of seconds^12^ requires sub-second data acquisition. Methods that are based on correlating intensity fluctuations such as fluorescence correlation spectroscopy (FCS) and image correlation spectroscopy (ICS) can achieve high acquisition rates but typically trade spatial resolution for temporal resolution or vice versa, as they require averaging of signal fluctuations for quantitative analysis^13^,^14^,^15^. Similarly, single-molecule localization-based super-resolution methods only achieve high spatial accuracy with slow acquisition rates and often require integration over long time periods for cluster detection^8^,^9^,^10^.

One technique that can measure membrane protein clustering with high spatial and temporal resolution is Förster resonance energy transfer (FRET). The temporal resolution of FRET is mainly limited by the acquisition rate of the camera or the scan speed in a laser-scanning microscope. FRET has an exquisite sensitivity as only molecules in close proximity (typically <10 nm) exhibit non-radiative energy transfer through dipole-dipole coupling. To detect FRET between proteins of the same species (with identical fluorophores) and thus protein self-association, so-called homo-FRET can be employed where the loss of anisotropy of the fluorescence emission is used as a read-out for FRET events^16^. Homo-FRET commonly makes the assumption that energy transfer to the acceptor results in depolarization. However, this assumption is not always valid for proteins fused to green fluorescent protein (GFP) because the rotational freedom of the fluorophores is restricted due to self-association^17^. Thus, homo-FRET can underestimate the degree of protein clustering. Alternatively, hetero-FRET has been used in the detection of protein clustering^18^,^19^,^20^. Here, a major concern is that the overall FRET efficiency of a given cluster is dictated by the ratio of donor and acceptor molecules in the cluster^19^,^21^, which can vary from cluster to cluster. Thus, it has been difficult to accurately measure protein clustering with FRET to date.

In the current study, we extended FRET to detect membrane protein clusters by the intermolecular associations of neighbouring proteins. Here the donor and acceptor are fused and expressed as a single-chain peptide so that the donor-to-acceptor ratio of 1:1 is fixed irrespective of the degree of clustering. In this construct, intramolecular FRET can also take place between the donor and acceptor on the same chain. In our experiments, we assumed that the distance and orientation between the two fluorophores within the sensor did not alter as a function of protein clustering. In this case, the efficiency of intramolecular FRET was similar for monomeric and clustered proteins. In contrast, intermolecular FRET efficiency between the neighbouring FRET pairs scaled with the distance between donors and acceptors and the number of acceptors present in the Förster radius of each donor molecule^21^,^22^,^23^. We named the sensor CliF (clustering reported by intermolecular FRET). It should be particularly applicable to membrane proteins where the rotational mobility of the proteins are restricted and only lateral interactions take place within the two-dimensional membrane.

As a proof-of-principle, we attached the CliF sensor to a light-sensitive photoreceptor cryptochrome 2 (Cy2), which oligomerizes upon irradiation with blue light^24^. This allowed us to monitor the dynamics of protein clustering in the plasma membrane in real time. Further, we monitored TCR clustering in resting and activated conditions, and followed the dynamics of TCR clustering on supported lipid bilayers as clusters form in the peripheral region of the immunological synapse and translocate to the centre^6^,^25^,^26^. Thus, the FRET sensor allowed us to link cluster remodelling to protein trafficking in the confined space of an immunological synapse.

## Results

### CliF exhibited enhanced FRET at high densities

**Figure 1:**
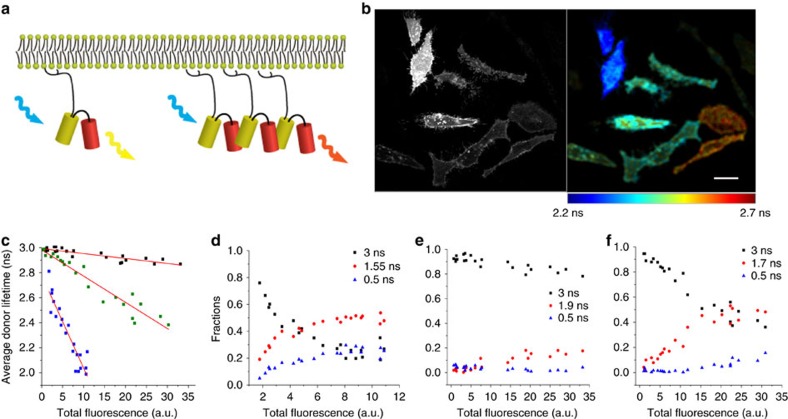
Lck10-CliF is sensitive to membrane protein concentration. (**a**) Schematic drawing of CliF fused to a membrane anchor (Lck10): the single-chain FRET pair Venus-mCherry can only exhibit intramolecular FRET as a monomer (left) and also intermolecular FRET in protein clusters (right). (**b**) Fluorescence intensity (left) and average donor lifetime image (right) of HeLa cells expressing Lck10-CliF. Lifetime values were pseudocoloured according to the colour scale. Scale bar, 10 μm. (**c**) Venus lifetime in Lck10-CliF (blue), Lck10-Venus only (black) and Lck10-Venus co-expressed with mCherry-H-Ras11 (green) as a function of total (donor+acceptor) intensity. Each data set was fitted to a straight line (red lines) yielding slopes of −0.075 (*R*^2^=0.866), −0.0039 (*R*^2^=0.886) and −0.021 (*R*^2^=0.925) for Lck10-CliF, Lck10-Venus and co-expressed Lck10-Venus and mCherry-H-Ras11, respectively. (**d**–**f**) Venus lifetime fractions as a function of total (donor+acceptor) intensity for Lck10-CliF (**d**), Lck10-Venus (**e**), and co-expressed Lck10-Venus and mCherry-H-Ras11 (**f**). Lifetime fractions were extracted by fitting the lifetime decay histograms of Venus to a three-component exponential decay function resulting in three distinct lifetime values, as indicated. Data in **c**–**f** were acquired for at least 30 cells per condition. More than 200 photons per pixel were recorded in the FLIM experiments.

To test whether protein clustering could be measured with a single-chain FRET pair, we fused the yellow fluorescent protein Venus with the red fluorescent protein mCherry. To avoid variations of intramolecular FRET efficiency due to flexibility in dipole alignment, we removed 11 amino acids from the C terminus of Venus and 7 amino acids from the N terminus of mCherry and limited the linker between the two fluorescent proteins to only serine-glycine. From here on, we call this construct CliF. We fused CliF with a membrane anchor (Lck10, the first 10 amino acid of Lck)^27^ that targets the protein to the plasma membrane (Fig. 1a,b). It is expected that at high expression levels of the sensor, FRET would also occur between the donors and acceptors of neighbouring sensors, that is, donors on one molecule would FRET with acceptors on a neighbouring molecule. Indeed, in transfected HeLa cells, cells with higher protein expression levels of Lck10-CliF consistently had a shorter donor fluorescence lifetime compared to cells with lower expressions (Fig. 1b). When we plotted the donor lifetime of CliF against the total cellular fluorescence (as a measure of protein concentration), we found a strong negative correlation (*R*^2^=0.866) that could be fitted to a line with a slope of −0.075. We constructed a control construct, Lck10-Venus that contained only the donor fused to the same membrane anchor. With this construct, Venus lifetimes were essentially independent of protein expression (*R*^2^=0.886, slope −0.0039, Fig. 1c). In contrast, co-expressing Lck10-Venus with mCherry-H-Ras11 (mCherry fused to the last 11 amino acids of H-Ras) in the same cell at equal quantities (by using a 2A peptide) resulted in a linear correlation with a slope of −0.021 (*R*^2^=0.925, Fig. 1c), indicating that the presence of mCherry-H-Ras11 at high density resulted in FRET with Lck10-Venus. The magnitude in the change in FRET efficiency as a function of acceptor concentration qualitatively matched the theoretical framework of non-interacting proteins in membranes described previously^28^,^29^. These data strongly indicate that the reduction in Venus lifetime in Lck10-CliF was caused by intermolecular FRET at high density. Notably, the Venus lifetime of CliF was three times more sensitive to expression level than co-expression of Lck10-Venus and mCherry-H-Ras11, highlighting the advantage of having the acceptor and donor fluorophore on the same protein.

Next, we examined the change in fluorescence lifetime of Venus in Lck10-CliF in more detail. To compare lifetime decay curves across experimental conditions, we pooled photons from entire images (>200,000 photons per curve) and tail-fitted the data to a triple exponential decay function (Supplementary Fig. 1). This model was empirically selected, as it was the least complex model that resulted in a good fit for all experimental conditions. We fixed the lifetime values at 3 ns, 1.55 ns and 0.5 ns (or left the intermediate lifetime free, Supplementary Fig. 2) and plotted the fractions against the expression level for each cell (Fig. 1d–f).

The 3 ns lifetime in Lck10-CliF is likely to represent the intrinsic lifetime of Venus in the absence of a functional acceptor as it was similar to the Venus lifetime in Lck10-Venus (Supplementary Fig. 1a) and the published value^30^. Non-functional mCherry acceptors in Lck10-CliF could be a result of a slow maturation rate and limited rotational freedom, and hence dipole alignment, particularly since the fluorescent proteins were membrane-bound^31^. The 3 ns lifetime fraction decreased with increase protein expression levels, suggesting the appearance of intermolecular FRET at higher densities. The reduction in the 3 ns lifetime fraction was accompanied by a raise in fractions for both the 1.55 and 0.5 ns lifetimes (Fig. 1d). The overall increase in FRET efficiency could be caused by enhanced intermolecular FRET through protein clustering and/or intramolecular FRET through better dipole alignment of the sensor in clusters.

In Lck10-Venus, the longest lifetime of ∼3 ns was clearly the dominant lifetime and its fraction decreased only slightly as expression levels of Lck10-Venus increased (Fig. 1e; Supplementary Fig. 2). In contrast, when Lck10-Venus and mCherry-H-Ras11 were co-expressed, the fraction belonging to the longest lifetime decreased and the fraction of the intermediate (1.7 ns) lifetime increased with increasing protein density (Fig. 1f; Supplementary Fig. 1c; Supplementary Fig. 2). The fraction of the shortest (0.5 ns) lifetime was constant and only slightly increased at extremely high expression levels. Taken together, we propose that the intermediate lifetime in Lck10-CliF could represented intermolecular FRET as it increased the most with protein density. The 0.5 ns lifetime in CliF may be regarded as Venus undergoing intramolecular FRET at low protein density conditions, as this fraction was very minor (<5%) with the donor-only construct even when mCherry-H-Ras11 was co-expressed. Lck10-CliF molecules under very high protein density conditions may also exhibit intermolecular FRET at very short lifetimes (that is, ∼0.5 ns). This is possible because as protein density increases, not only the fraction of molecules engaged in intermolecular FRET increases, the efficiency of intermolecular FRET itself also increases markedly as more acceptor molecules are available for energy transfer per each donor molecule. The slight gain of the intermediate lifetime under high protein density in Lck10-Venus was likely due to change in chromophore conformation under protein crowding conditions as recently reported^32^. It should note that the change in amplitude of 1.9 ns in Lck10-Venus was substantially different from the change of 1.55 ns in CliF both in intensity and rate as a function of protein density. It is likely that the FRETing events in CliF override the change of chromophore conformation under protein crowding conditions.

### CliF reported light-induced protein clustering

**Figure 2:**
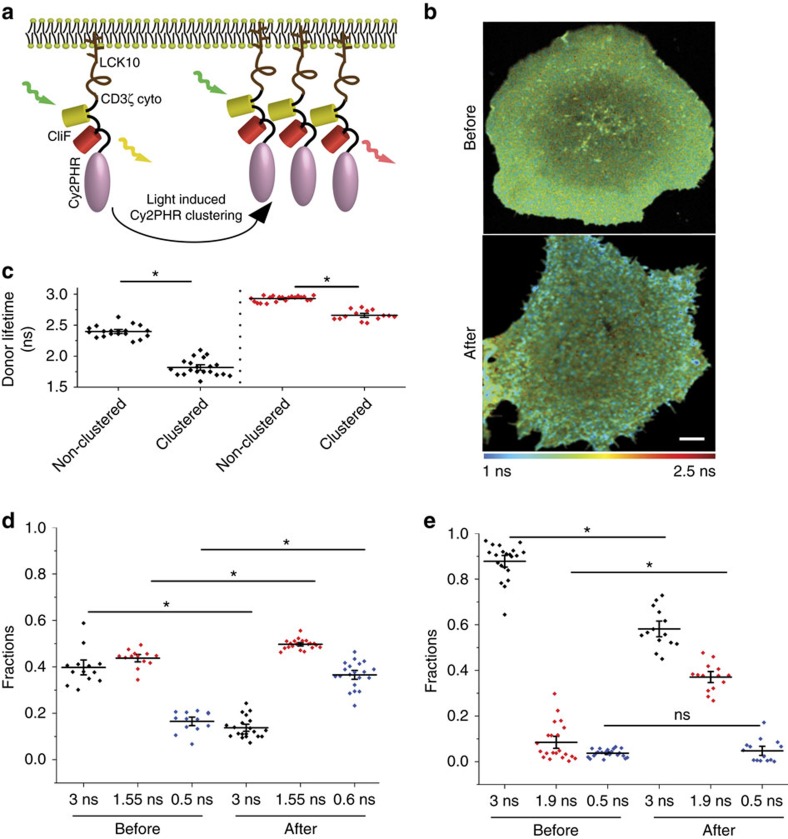
FLIM analysis of the donor fluorescence in CY2PHR-CliF. (**a**) Schematic drawing of CY2PHR-CliF consisting of a membrane anchor (Lck10), the cytosolic tail of CD3ζ (CD3ζ_cyto_), CliF and CY2PHR. Upon irradiation with light, CY2PHR-CliF forms clusters, leading to an enhanced intermolecular FRET sensed by CliF. (**b**) Intensity-weighted, pseudocoloured FLIM image of Venus (coloured blue to red for short to long lifetimes, as indicated) in COS-7 cells expressing CY2PHR-CliF before and after 30 s irradiation with low intensity 488 nm light to induce clustering. Scale bar, 2 μm. (**c**) Average donor lifetime of CY2PHR-CliF (black) and CY2PHR-Venus (red) in transfected cells before and after 488 nm light irradiation, that is, under non-clustered and clustered conditions. Horizontal bars indicate means (*n*=20 cells), error bars are s.e.m.; *P<0.01 (unpaired *t*-test). (**d**,**e**) Venus lifetime fractions in CY2PHR-CliF (**d**) and CY2PHR-Venus (**e**) before and after light-induced clustering. The fractions were obtained by fitting Venus lifetime intensity decays to a triple exponential decay function with the indicated lifetime values. Horizontal bars indicate means and error bars are s.e.m. Data shown in **d**,**e** are from at least 15 cells, *P<0.01 (unpaired *t*-test).

Given the sensitivity of Lck10-CliF to protein concentrations in the plasma membrane, we were motivated to test the possibility of CliF detecting the formation of membrane protein clusters. Thus, we fused CliF to a membrane protein whose self-association can be artificially controlled. We used the light-sensitive domain CY2PHR (amino acids 1–498) of the photoreceptor Cryptochrome cloned from *Arabidopsis*, which exhibits reversible light-dependent clustering^33^. As before, we also incorporated a membrane anchor^27^ to target the protein to the plasma membrane. While a construct lacking the membrane anchor displayed normal light-induced clustering properties (data not shown), the initial membrane-anchored fusion proteins of CY2PHR failed to form clusters. Thus, we inserted the cytoplasmic domain of CD3ζ (∼100 amino acids) of the TCR–CD3 complex between the membrane anchor (Lck10) and CliF (Fig. 2a) to increase the distance of CY2PHR to the plasma membrane, which fully recovered the light-inducible clustering properties of CY2PHR. The resulting CY2PHR-CliF reporter not only localized to the plasma membrane in COS-7 cells but also clustered upon irradiation with 488 nm light (Supplementary Movie 1).

We performed donor lifetime imaging with CY2PHR-CliF prior and post irradiation with 488 nm light. As a control, we also examined the lifetime changes of a donor-only construct, CY2PHR-Venus, under the same conditions. It was expected that light-induced CY2PHR-CliF clustering would increase the non-radiation energy transfer and depopulate the excited state of the donor molecules, resulting in a shortening of its lifetime. The FLIM imaging of CY2PHR-CliF expressed in COS-7 cells showed that the clustered region displayed a reduced lifetime, which was substantially shorter than the lifetime of non-clustered regions within the same image or before irradiation (Fig. 2b). The lifetime of Venus in CY2PHR-CliF was reduced on average from 2.41 ns to 1.82 ns due to clustering (Fig. 2c), representing a 32.9% change. When the donor-only construct CY2PHR-Venus was examined under the same condition, we also found a reduction in fluorescence lifetime from 2.95 ns to 2.69 ns. We attributed this 10% change in lifetime to the recently reported change in chromophore conformation due to molecular crowding^32^. We ruled out the possibility of photobleaching-induced lifetime changes^34^,^35^ in our experiments (Supplementary Fig. 3) but there could be other environmental factors that can impact on fluorescence lifetimes. Nevertheless, we concluded that the acceptor in CY2PHR-CliF substantially increased the sensitivity of the Venus lifetime to neighbouring molecules and thus the dynamic range of the cluster sensor.

As with the experiments shown in Fig. 1, Venus lifetime in the CY2PHR-CliF was also best fitted to a triple exponential decay function with lifetimes of 3 ns, 1.55 ns and 0.5 ns for the non-clustered CY2PHR-CliF and 3 ns, 1.55 ns and 0.6 ns for the clustered CliF (Supplementary Fig. 4a). When the intermediate lifetime were left free, the fitting returned an intermediate lifetime of 1.74±0.25 ns and 1.56±0.29 ns (mean±s.e.m.) before and after clustering, respectively, and the fractions were comparable with a fixed and free lifetime (Supplementary Fig. 5a,c). This suggests that changes in Venus lifetime induced by clustering (Fig. 2b,c) and high protein density (Fig. 1) are likely to be caused by the same mechanisms. Fitting of the Venus-only CY2PHR-Venus construct to a triple-component decay function resulted in lifetime values of 3 ns, 1.9 ns and 0.5 ns (Supplementary Fig. 4b) and 3 ns and 1.15±0.37 ns before light-induced clustering, and 3 ns and 1.71±0.20 ns after clustering when fitted to a double-exponential decay function (Supplementary Fig. 5b,d). We compared the fractions belonging to these lifetimes for both CY2PHR-CliF (Fig. 2d) and CY2PHR-Venus (Fig. 2e). As anticipated, the fraction of the 3 ns lifetime in CY2PHR-CliF that we assigned above to the intrinsic Venus lifetime was reduced upon light-induced clustering due to increased FRET. There was a concomitant small increase in the 1.55 ns fraction and a substantial increase in 0.6 ns fraction. One possible explanation is that the 1.55 ns lifetime represents CY2PHR-CliF molecules located in small clusters that had limited capacity for intermolecular FRET, while the 0.6 ns lifetime reflects CY2PHR-CliF molecules positioned in bigger and denser clusters, where the donor molecules could transfer a substantial amount of energy to nearby acceptor molecules. In this scenario, the fraction of 0.6 ns lifetime would include both molecules that undergo intramolecular FRET as well as molecules that undergo intermolecular FRET in very dense protein clusters. This is because when the excited donor molecule is surrounded by multiple acceptors in close proximity, the probability of the donor molecule transferring its energy to the nearby acceptor molecules increases linearly with the number of acceptors^21^,^22^, which would result in additional shortening of the lifetime and could account for a fractional change between the 1.55 ns to 0.6 ns lifetimes. However, it should be noted that other environmental factors can also impact on the fluorescence lifetime of fluorescent proteins. In CY2PHR-Venus, the fraction of the 3 ns lifetime also reduced upon clustering with a concurrent increase in the 1.9 ns lifetime fraction, which is likely due to protein crowding-induced chromophore change that has recently been described^32^. This suggests that Venus itself is sensitive to protein clustering. In conclusion, although the exact mechanism of how protein clustering impacts on the Venus lifetime is not fully understood, it does not preclude the use of CliF as a qualitative cluster sensor.

**Figure 3:**
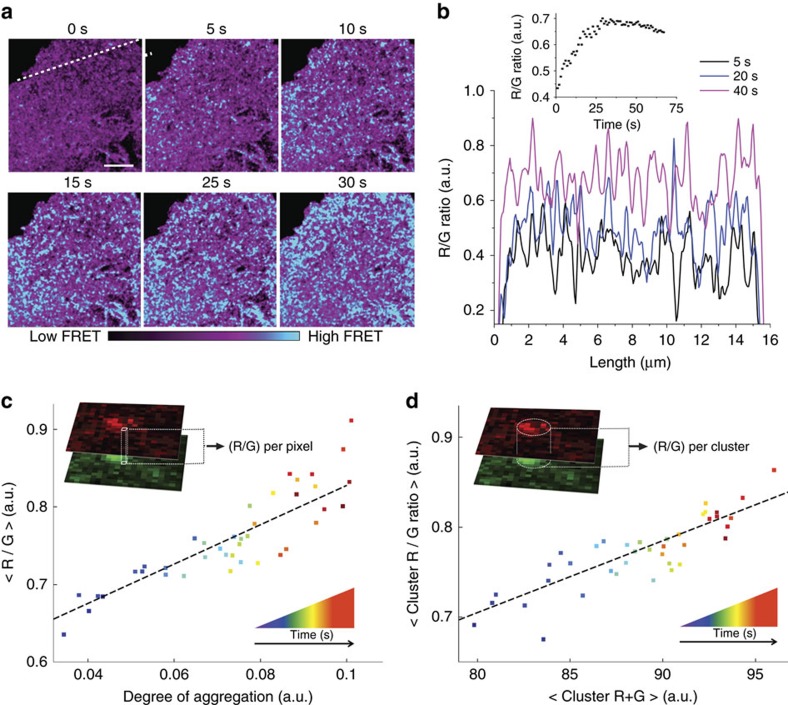
Detection of light-induced CY2PHR clustering by CliF with image correlation spectroscopy (ICS) and particle tracking. (**a**) Donor/acceptor (R/G) ratiometric images of light-induced clustering of CY2PHR-CliF in transfected COS-7 cells at the indicated time points. Images are pseudocoloured from purple to blue corresponding to low and high FRET efficiency. Scale bar, 2 μm. (**b**) R/G values along line scans at positions shown in **a** at selected time points. Inset: R/G ratio of individual frames plotted as a function of time. (**c**) Image Correlation Spectroscopy (ICS) analysis of data shown in **a**. Scatter plot of R/G ratio against degree of aggregation for each frame. The amplitude of ICS was normalized to the total intensity (donor+acceptor intensity, R+G) for each frame yielding a value for the degree of aggregation (*x* axis). It displayed a linear relationship (slope=2.52, *R*^2^=0.75) with CY2PHR-CliF FRET efficiency (R/G ratio, *y* axis). (**d**) Cluster tracking of data shown in **a** for each cluster, the R/G value (reflecting cluster density) was plotted against the R+G value (reflecting number of molecules), resulting in a linear relationship (slope=0.008, with *R*^2^=0.72). Symbols are coloured in **c**,**d** according to the frame number during the time course. Data in **b**–**d** is single representative data of 5 cells.

We next tested the performance of CliF to monitor clustering of CY2PHR in live cells, using a simple ratiometric imaging approach that does not require fitting. Here, we employed a 514 nm laser for both light-induced clustering and excitation of Venus. We recorded the intensity ratio of donor (525–555 nm) and acceptor (610–700 nm) fluorescence (R/G ratio) as a measure of FRET efficiency by laser-scanning confocal microscope at frame rate of 1 Hz. Upon the irradiation with 514 nm light, optically resolved clusters started to appear at the cell surface (Fig. 3a). The formation and growth of clusters could be seen in line profiles taken at different time points (Fig. 3b), where emerging clusters had a high FRET efficiency (R/G ratio). Although the position of the clusters shifted slightly over time, indicating that clusters were mobile, the increase in FRET efficiency of individual clusters could be observed as the clusters grew in size. When followed over the entire image, the FRET efficiency reached a plateau ∼40 s after light-induced clustering (Fig. 3b, insert). Compared to the non-clustered R/G ratio at time 0 s, the FRET efficiency increased by 63%. This result clearly demonstrated the ability of CliF to detect membrane protein clustering by ratiometric imaging with high spatial and temporal resolutions.

Next, we assessed whether the CliF could be multiplexed with analysis such as image correlation spectroscopy (ICS) and single-particle tracking (SPT) to quantify the spatiotemporal organization of the tagged protein. In ICS, the amplitude of the spatial correlation function is inversely proportional to the number of features that produce intensity fluctuations in the image^13^. As the molecules become more clustered, the number of fluctuating features was reduced, whereas the total intensity of the image stayed constant regardless of the clustering state of the molecules (Supplementary Fig. 6). Therefore, the ratio of the amplitude of the ICS correlation function to the image intensity reflected the average density of the clusters, termed the degree of aggregation. As expected, the degree of aggregation increased for CY2PHR-CliF over time (Fig. 3c). When the degree of aggregation of each frame was plotted against the FRET efficiency in these images (R/G ratio), we found a positive linear correlation, suggesting that the overall CliF FRET efficiency method faithfully reports protein clustering.

We also used a single-particle tracking algorithm to map the centre of individual clusters. This was done by fitting the sum of donor and acceptor (R+G) intensity profiles of each cluster to a Gaussian function, thus extracting the amplitudes of R/G and R+G for each cluster. As clustering increased over time for the CY2PHR-CliF protein, the R/G value reflecting the density of the cluster was expected to increase with time. Similarly, the R+G value of individual clusters that can be equated to the number of molecules per cluster also increased over time since CY2PHR-CliF clusters formed by aggregation of monomers. We found the expected positive linear relationship between R/G and R+G values for individual clusters (Fig. 3d). Taken together, the experiments with CY2PHR-CliF demonstrate that CliF reports protein clustering and can be paired with established approaches for spatiotemporal analyses of molecular dynamics.

### Detection of T cell receptor clusters by CliF

We used the developed CliF to dynamically measure the density of TCR clusters. Indeed, the packing density of TCR–CD3 complexes is currently not known. This is important because the density of TCR clusters could reflect their signalling state. In resting cells, the cytoplasmic domains of CD3ζ and CD3ɛ are bound to the inner leaflet of the plasma membrane through electrostatic interactions, making them inaccessible to the kinase Lck and thus phosphorylation^36^,^37^. At high TCR–CD3 density, the cytoplasmic tails of CD3ζ and CD3ɛ may detached, allowing TCR signalling to begin. Thus, TCR–CD3 clustering may render the receptor the signal competent.

**Figure 4:**
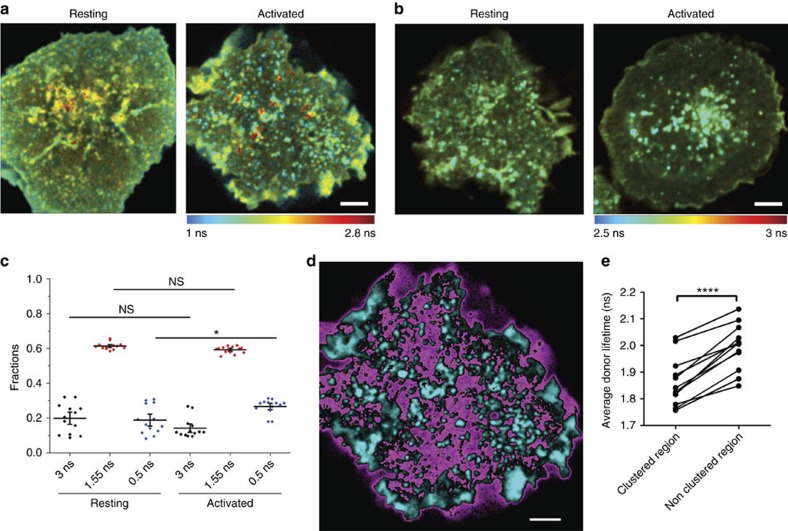
FLIM imaging of CD3ζ-CliF clustering in T cells. (**a**,**b**) Donor lifetime image of Jurkat cells expressing CD3ζ-CliF (**a**) or CD3ζ-Venus (**b**) under resting (glass surface coated with anti-CD90 antibodies) and activating (glass surface coated with a mixture of anti-CD3 and anti-CD28 antibodies) conditions. The lifetime is pseudocoloured blue to red as indicated by the colour scale. Scale bar, 2 μm. (**c**) Lifetime fractions derived from triple exponential decay fits in resting and activated T cells. Horizontal bars indicate means and error bars are s.e.m. Data are from *n*=13 cells per condition. (**d**,**e**) Comparison of average donor lifetime of high-intensity regions (cyan, based on pixel intensity) and low-intensity regions (magenta) (**d**) of the activated cell shown in **a** and corresponding values (**e**). In **e**, each symbol is one cell, *P*<0.01, *n*=9 cells (paired *t*-test). More than 200 photons/pixel were recorded for all FLIM data.

First, we wanted to test whether CliF could be used to detect TCR clusters. To do so, we fused CliF to the C terminus of full-length CD3ζ and used a donor-only construct, CD3ζ-Venus, as the control. Endogenous and over-expressed full-length CD3ζ forms homodimers and is constitutively associated with the TCR–CD3 complex^38^. We stimulated Jurkat cells expressing either CD3ζ-CliF or CD3ζ-Venus on glass coverslips coated with anti-CD90 (non-activating) or anti-CD3 and anti-CD28 antibodies (anti-CD3+CD28 Ab, activating) to compare resting and activated T cells. We verified that anti-CD90 surfaces did not cause intracellular calcium fluxes and CD69 expression when compared to cells on non-functionalized, poly-L-lysine (PLL) coated coverslips and cells in suspension (Supplementary Fig. 7). Visual inspection of the donor lifetime image suggests that CD3ζ-CliF and CD3ζ-Venus formed clusters in resting cells, and that the clustered CD3ζ-CliF exhibited a shorter lifetime compared to the non-clustered CD3ζ-CliF (Fig. 4a,b). The average donor lifetime was 2.01±0.05 ns and 1.86±0.04 ns (mean±s.e.m.) for CD3ζ-CliF, and 2.91±0.01 ns and 2.85±0.02 ns for CD3ζ-Venus in resting and activated T cells, respectively. Thus, a greater change in lifetime was observed for CD3ζ-CliF than CD3ζ-Venus upon T-cell activation, resulting in a range of FRET values for CD3ζ-CliF in activated T cells (Fig. 4a). The heterogeneity in FRET efficiency with CD3ζ-CliF in clusters in the same image suggests that clusters had a variable molecular density, which agrees with single-molecule localization microscopy data^4^.

To provide more details of how Venus lifetimes differed in resting and activated T cells, we again fitted Venus lifetime decay to a triple exponential decay function with lifetime values of 3 ns, 1.55 ns and 0.5 ns. The lifetime fractions suggest that the majority of CD3ζ-CliF molecules resided in clusters that underwent intermolecular FRET with lifetime of 1.55 ns (Fig. 4c). An alternative fitting procedure where the intermediate lifetime in the triple exponential decay function was left free produced similar results (Supplementary Fig. 8). Following the interpretation of the lifetime fraction of CY2PHR-CliF (Fig. 2d), CD3ζ-CliF molecules undergoing intermolecular FRET could have a lifetime of 1.55 ns and 0.5 ns. There were no statistically significant changes with T-cell activation in the fraction of the 1.55 ns lifetime. One possible explanation is that the fraction and density of the less dense and smaller clusters of CD3ζ-CliF were not remodelled following activation (Fig. 4c). In contrast, a significant increase in the fraction corresponding to the 0.5 ns lifetime was observed, suggesting that CD3ζ-CliF molecules formed larger and more compacted clusters upon T-cell activation. However, since the origin of the 0.5 ns fraction is not completely understood, other explanation of how CD3ζ-CliF clustering impacts on the observed lifetime changes may also be possible.

Next, we thresholded the FLIM image based on the intensity of individual pixels to distinguish non-clustered (magenta) from clustered (cyan, Fig. 4d) regions. We used the average photon arrival time as a measure of mean lifetime. This revealed that the clustered region indeed had a shorter lifetime than the non-clustered region in all the cells (Fig. 4e). In summary, we demonstrated that the attachment of CliF to CD3ζ of the TCR complex was sufficient to detect TCR clusters. The result implicates a remodelling of TCR cluster density upon T-cell activation.

To extract quantitative information from these data, for example the maximum number of acceptors a CD3ζ-CliF molecule could transfer its energy to and the Förster radius at maximum FRET efficiency (see Supplementary Note, Supplementary Figs 9 and 10), requires the assumptions that (i) lifetime changes are caused by FRET and not other environmental factors, (ii) intramolecular FRET does not change with clustering and (iii) that the intermolecular transfer rate depends only on the number of other CliF molecules within the cluster. As discussed above, it is challenging to unequivocally and exclusively assign changes in donor fluorescent lifetime to physical processes such as intermolecular and intramolecular FRET. Quantitative analysis of FRET data with fluorescent proteins is particularly difficult given that many fluorescent proteins do not have monoexponential decays and that even randomly orientated fluorescent proteins undergoing FRET can exhibit different FRET efficiencies although only one FRET process is present^39^. The advantage of CliF, and the reason for designing it, lays in its ability to qualitatively record the spatiotemporal variation of clustering of membrane proteins with high sensitivity. Thus we next examined CD3ζ clustering in live T cells.

### TCR clustering and movement in the immune synpase

Signalling TCR microclusters are predominantly formed at the outer region of the immunological synapse^5^,^6^,^7^ and engaged TCR are preferentially transported to the centre of the immunological synapse^40^. This active, actin-mediated movement is thought to regulate TCR signalling^6^,^41^. Hence, we made use of the live-cell imaging capability of CliF to determine how the density of TCR clusters is modulated during its centripetal transportation from the periphery to the central region of the immunological synapse, referred to as the peripheral and central super-molecular activation cluster (cSMAC and pSMAC, respectively). We used supported lipid bilayers that contained the adhesion protein ICAM-1 and peptide-presenting major histocompatibility complex class-I (pMHC-I) molecules to stimulate Jurkat cells expressing the cognate TCR OT-I. When T cells are triggered with pMHC molecules on laterally mobile bilayers, TCR clusters form at the periphery and are transported to the cSMAC in an actin-dependent manner^25^. Here we tracked cluster movement and density with CD3ζ-CliF and found that CD3ζ-CliF clusters had a higher velocity of both diffusion and net flow in T cells on bilayers compared to CD3ζ-CliF clusters in T cells on antibody (anti-CD3+anti-CD28 Ab)-coated coverslips (Supplementary Fig. 11). The observed diffusion values of TCR were similar to previous reports^7^,^42^.

**Figure 5:**
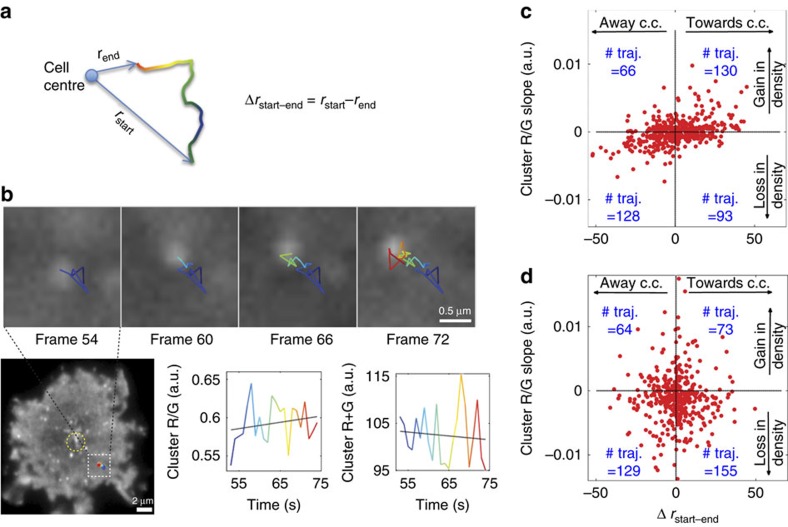
Tracking TCR cluster movement and density during T-cell activation. (**a**) Tracking analysis to determine directionality of CD3ζ-CliF clusters: Δ*r*_start-end_ measures cluster movement towards or away from the cell centre. The trajectory is colour-coded from blue to red to indicate positions over time. (**b**) Representative trajectory of a CD3ζ-CliF cluster (white dotted square in cell image) some distance from the cell centre (yellow circle in cell image) and the resulting R/G and R+G traces for this trajectory. The slopes of the R/G and R+G trajectories were extracted (black line) by linear regression. Scale bar, 2 μm, and 0.5 μm in the zoomed in images in **b**. (**c**,**d**) Scatter plot of the R/G slopes versus the Δ*r*_start-end_ values of ∼400 TCR trajectories for cells activated on pMHC-presenting lipid bilayers (**c**) and antibody (anti-CD3+anti-CD28 Ab)-coated surfaces (**d**). Scatter plots were divided into four quadrants, corresponding to gain/loss of cluster density and movement to/away from cell centre (c.c.). The number of trajectories in each quadrant is stated. Data shown in **c**,**d** is analysis from a single cell. 5 cells were analysed under each conditions.

The two-channel time-lapse imaging under TIRFM showed that cells begun to spread on the bilayer about 1 min after landing. TCR clusters were continuously formed at the cell edge and transferred gradually to the cell centre as previously described^5^,^6^,^7^. We employed single-molecule tracking algorithms^43^ to track individual clusters over time (Fig. 5a,b). As above, we tracked R/G and R+G values for each point in the trajectory. While both values are proportional to the number of molecules contained in the clusters, the ratiometric measurement is less influenced by noise, loss of focus and local variation of protein concentration due to membrane ruffling. Even in individual trajectories, R+G plots frequently displayed more variations over the length of the trajectory than R/G plots (Fig. 5b).

To map the change in cluster density as clusters translocate towards the cell centre, the change in distance of the cluster relative to the cell centre, Δ*r*_start-end_, was defined as illustrated in Fig. 5a. The R/G slope of each trajectory was plotted against the Δ*r*_start-end_ value so that each trajectory fell into one of four quadrants: moving towards versus away from the cell centre and gaining versus losing in cluster density (Fig. 5c,d). For the T cells stimulated with mobile pMHC molecules there was a gain in cluster density as the TCR clusters moved towards cSMAC at the cell centre, and there was a loss of cluster density as TCR moved away from cell centre (Fig. 5c). No such trend was found for T cells stimulated with adherent immobilized antibodies (Fig. 5d). Rather, a net loss of CD3ζ cluster density in the antibody-stimulated cells was observed. There were 284 out of 421 trajectories displayed a decrease in cluster density. This suggests that trafficking of TCR–CD3ζ clusters through the immunological synapse towards the cSMAC is associated with remodelling into denser aggregates. These experiments further highlight the strength of CliF as a clustering sensor to report the dynamic properties of protein clusters under live-cell conditions.

## Discussion

Protein clustering in live cells can be monitored by time-resolved anisotropy measurements of homo-FRET. Because anisotropy can be influenced by the unknown constrains of the rotational freedom of the fluorophores^16^,^17^, we were motivated to develop a membrane-anchored, single-chain FRET pairs that underwent intermolecular FRET at high molecular density and called the resulting cluster sensor CliF. CliF utilized the sensitivity of FRET to detect membrane protein clusters irrespectively of the presence of non-clustered molecules. CliF can easily be implemented in conjunction with commonly used microscopy setups such as wide-field and confocal microscopes, and lifetime changes monitored via FLIM. Further, CliF can be multiplexed with analyses such as SPT and ICS.

Thus, the sensor can be used to monitor the rate of protein clustering and the movement of clusters within the plasma membrane. It should be noted that donor–donor and acceptor-acceptor homo-FRET might also occurred when CliF molecules were closely spaced. Here one would anticipate that homo-FRET in CliF is bidirectional and occurs in an oscillating manner, resulting in little net energy loss. Thus, we assigned changes in FRET efficiency to intermolecular FRET did not formally rule out other possibilities. Given that Venus, the fluorescent molecule that we used as a donor, is itself sensitive to molecular crowding^32^, the sensor may be improved by changing the pair of fluorescent proteins.

We applied the sensor to TCR clustering, correlating cluster movement to cluster remodelling within the immunological synapse. Previous reports identifying and tracking TCR clusters based solely on local intensity measurements may have missed subtle rearrangements and are limited by the number of clusters that can be analysed per cell^7^,^42^. A particularly attractive feature of the CliF sensor is that it could be used to track CD3ζ clusters in the immunological synapse. We compared tracking based on intensity versus density and found the latter to have reduced variation within and between trajectories. While it is known that TCR clusters continuously form and are actively transported to the central region of the immunological synapse in an actin-dependent manner^25^,^26^, whether and how clusters are remodelled during the transport has been difficult to map out. Using CliF to track CD3ζ clusters, we found a dramatic difference in the remodelling of CD3ζ cluster densities with the TCR stimulation method: while in T cells on pMHC-containing bilayers, clusters that moved to the centre of the synapse increased in density, clusters in T cells stimulated with adherent antibodies predominantly decreased in density. The differences in cluster remodelling with the two methods of T-cell stimulation may account for the differences in the temporal evolution of signalling as well as the sustainability of signalling. In conclusion, we designed an intermolecular FRET sensor that maps clustering of membrane proteins in space and time that may be used to provide insights into how different molecular densities impact on signalling processes.

## Methods

### DNA constructs

The DNA oligonucleotides used in the project were purchased from Integrated DNA Technology Australia. Primer sequences used are provided in Supplementary Table 1. The CY2, Venus and mCherry DNA constructs were purchased from Addgene. Q5 DNA polymerase (New England BioLabs) was used for all PCR reactions. Standard PCR and restriction enzyme cutting, or overlapping extension PCR methods were conducted for the cloning procedures. The constructed plasmid sequences were confirmed by BigDye Terminator v3.1 Cycle Sequencing (Life Technologies) at the Ramaciotti Centre for Genomics at University of New South Wales.

### Cell culture and transfection

COS-7 and HeLa cells were cultured in DMEM supplemented with 10% Foetal Bovine Serum (FBS). Jurkat E6.1 and the OT-I T-cell clones were cultured in Roswell Park Memorial Institute medium (RPMI) supplemented with Glutamine and10% FB. All cells were cultured in 37 °C incubators with 5% CO_2_. Adherent cells were transfected by Lipofectamine LTX (Invitrogen) 18 h before imaging in glass bottom Fluorodish cell culture dishes (Coherent Scientific). T cells were transfected using a microporator (Invitrogen Neon) following the manufacturer's protocols. The culture medium was exchanged for HEPES-buffered and phenol red free medium before imaging. All cell lines used were tested and found negative for *Mycoplasma* contamination.

### Flow cytometry

After been incubated on the indicated surface for 12 h, 10^6^ Jurkat cells were washed and incubated with anti-human CD69 Alexa Fluor488 (310916, BioLegend) at 4 °C for 30 min. Cells were subsequently washed and resuspended in 300 μl buffer and analysed using BD bioscience FACSanto II Flow cytometer. A total of 20,000 evens were acquired for each sample and the data were analysed using FlowJo software (TreeStar, Ashland, OR) by gating for positive and negative CD69 expression based on negative control samples.

### Preparation of lipid bilayers and membrane sheets

Supported lipid bilayers (SLB) were prepared from a lipid mixture containing 97 mol% DOPC (1,2-dioleoyl-sn-glycero-3-phosphocholine), 2 mol % DGS-NTA (Ni) (1,2-dioleoyl-*sn*-glycero-3-[(*N*-(5-amino-1-carboxypentyl) iminodiacetic acid) succinyl]) and 1 mol % Biotin-PE (1,2-dioleoyl-*sn*-glycero-3-phosphoethanolamine-*N*-(cap biotinyl)) (Avanti Lipids). The mixture was dried under a nitrogen flow to remove chloroform. The dried lipid film was rehydrated in 10 mM HEPES buffer containing 150 mM NaCl, 100 μM EDTA, and sonicated using a tip sonicator (Sonifier 250, Branson) on ice until clear. The liposome solution was filtered through 0.22 μm filter and added to plasma cleaned cover glass (24 × 50 mm, 0.17 mm thick, Menzel-Gläser) attached to the bottom of 8 well Lab-Tek chambers (Nunc) using silicone based glue (Dubsil 22, Ultimate Dental). CaCl_2_ (2 mM final concentration) was added to the lipid mixture to initialize formation of SLB. After washing with PBS (Ca^2+^ and Mg^2+^ free), the SLB were coated with streptavidin (Sigma Aldrich; 2 μg ml^−l^) and NiCl_2_ (100 μM) in PBS for 10 min. The SLB were subsequently washed with PBS and incubated with monobiotinylated pMHC (500 ng ml^−1^) and His-tagged ICAM-1 (250 ng ml^−l^) for 1 h at room temperature.

Membrane sheets were prepared following a previously described method^44^. Briefly, the cell medium was exchanged to 10 mM Ca^2+^ and Mg^2+^ free PBS for 30 s. Cells swelled under these conditions due to high cytosolic osmotic pressure. A PLL-coated coverslip, glued to the opening of a 1.5 ml microcentrifuge tube, was placed on top of the cells, and gentle pressure was applied to ensure that the PLL-coated coverslip made contact with the cell membrane. After 2 min, the microcentrifuge tube with the PLL-coated coverslip was quickly lifted, which produced a ‘pop' sound. This procedure results in the apical section of the plasma membrane being removed while the basolateral section of the membrane remained attached to the substratum. The membrane sheet was washed gently three times in 10 mM Ca^2+^- and Mg^2+^-free PBS before addition of CaCl_2_.

### Imaging and image correlation spectroscopy

All the ratiometric imaging experiments (except OT-I T- cell activation on SLB) were performed on a Leica TCS SP5 microscope. A 63 × objective lens (numerical aperture (NA)=1.4, oil) was used for all image acquisition. For ratiometric FRET imaging, the CW 488 nm Argon laser was used, where the emission was gated at 500–550 nm and 610–690 nm for donor and acceptor detection, respectively. The Leica hybrid detector with high detection efficiency and line averaging was used for higher signal-to-noise ratio. For high-speed imaging, the resonance scanner with scanning frequency of 8 kHz was used. The confocal pinhole size was set to 95 μm. Laser power and detector gain were adjusted so that no pixel saturation occurs. For the calculation of FRET efficiency from acceptor to donor ratiometric image, we used


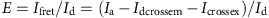


that operated at pixel basis, where *I*_fret_ refers to FRET intensity, *I*_d_ refers to donor intensity in the absence of acceptor, *I*_a_ is the acceptor intensity in the presence of donor, *I*_dcross em_ is the fraction of donor emission cross-emitted into the acceptor channel and *I*_cross ex_ is the fraction of acceptor intensity cross-excited by donor excitation.

T-cell activation on SLB was imaged on the Zeiss Elyra PS.1 microscope in Total Internal Reflection Fluorescence (TIRF) mode with two-channel acquisition. A 488 nm CW Argon laser was used for excitation through Zeiss × 100 objective (NA=1.57, oil). The emitted fluorescence was split by a 560 nm beam splitter and further purified by two band pass filters at 495–550 and 570–620 nm, respectively. Two cooled EMCCD cameras (Andor Ixon 897) with 512 × 512 image format and pixel size of 100 nm were used for image acquisition at a frame rate of 2 Hz.

For Ca^2+^ imaging of T cells on resting and activated surface, cells were loaded with Fluo4, incubated on the indicated surface for 15 min and fixed with PFA. Fluo4 was excited with a 488 nm Argon laser and fluorescence collected between 500 and 550 nm. The differential interference contrast DIC images were acquired simultaneously by illuminating the cells with a 633 nm laser and collecting the transmitted light with a camera.

To confirm that the level of protein aggregation of CY2PHR-CliF reported by the FRET signal of CliF is valid, we analysed the data by image correlation spectroscopy (ICS) and derived the degree of aggregation. This approach is used to assess the average level of aggregation from time evolving image series (Supplementary Fig. 6a), and was previously applied to several systems^45^,^46^. The technique consists in calculating the spatial image auto-correlation function (ACF) for every frame in the time series (Supplementary Fig. 6c). As the proteins aggregate over time, the average intensity of an image in the series does not change (Supplementary Fig. 6b), given that there is no significant photobleaching and there is no net change in protein concentration due to the endo- or exocytosis, during the acquisition time. On the other hand, ACF increases in amplitude with time, due to the decrease in the number of features present in the region of interest (Supplementary Fig. 6c). The ratio of average image intensity and inverse of the ACF amplitude, g(0,0), defines the degree of aggregation for every time point (Supplementary Fig. 6b). The ACF and degree of aggregation were calculated as previously described^45^,^46^ in a custom script written in Matlab (Natick, MA).

### Single particle tracking

To extract the FRET signal of CY2PHR and TCR clusters, the time series of R/G was subjected to single-particle tracking analysis using the GUI interface of Diatrack software^43^. The data were pre-processed using a Gaussian smoothing filter with a width at the half maximum equal to 3.4 pixels. The low-intensity features were filtered using an intensity threshold of 100 (out of 255 maximum). The position of the centre of the cluster was extracted by fitting the cluster intensity profile to a Gaussian function. The spatial coordinates of the clusters were recorded and fed back into the time series data to extract the R/G and R+G values of the clusters. To avoid the inaccuracy of the fitting in low signal-to-noise conditions, the average of the surrounding 8 pixels of the central coordinates was taken for both R/G and R+G values at every time point where the cluster was detected. For the tracking, the option ‘toggle' of the Diatrack package was used to estimate the maximum spatial jump a cluster was allowed to make between subsequent frames. It was found to be about 10 pixels, and this value was used for processing of all image sets. The final tracks were gap closed with the option ‘Analysis>Trajectory pre-processing>close gap in trajectories' to ensure that all the short trajectories with likely connectivities were merged together. The trajectories' statistics were calculated by custom written scripts in Matlab (Natick, MA). When the R/G or R+G values of the trajectory were plotted as a function of time, a line of best fit was extracted by linear regression analysis. The slope of the extracted line was used to assess the gain and loss of the detected cluster density. If the slope was positive, the cluster was considered to be gaining in molecular density, while negative slope implied a decrease in the cluster molecular density.

The net flow and diffusion coefficient of the cluster were obtained by fitting the mean squared displacement (MSD) curve of each trajectory using the quadratic equation: 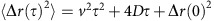 where the last term on the right hand side describes, zero temporal lag offset, usually attributed to camera noise. The first term of this equation describes the quadratic relationship of the clusters net flow with the temporal lag, while the second term describes the diffusion coefficient of the cluster. If a cluster exhibits a net flow towards or away from cell centre, then v will have a non-zero value and D will also take on small values due to lateral diffusivity of the cluster. On the other hand, clusters exhibiting predominantly diffusive behaviour, will have a finite value for D and while v will equal to zero. We grouped all the values of D and v into histograms for both conditions of T-cell activation on antibodies coated surfaces and lipid bilayers (Supplementary Fig. 11).

### FLIM-FRET imaging and analysis

The donor lifetime imaging was conducted on a Leica TCS SP5 that is coupled with a pulsed white light laser and MPD (Picoquant, Germany) detector. The TimeHarp260 (Picoquant, Germany) was used as a timing device to measure the time delay between excitation and photon arrival. For excitation, the white light laser was tuned to 488 nm and pulsing at 40 MHz. The emitted fluorescence was guided to the external port of the Leica microscope and split by a 560 nm beam splitter. The donor fluorescence was reflected and further purified by a 500–550 nm band pass filter before its arrival to the SPAD detector. The acceptor fluorescence was collected by a second SPAD detector through a 580–650 nm band pass filter. The Time Correlated Single Photon Counting TCSPC histogram was reconstructed from the Time-Tagged Time-Resolved TTTR data at pixel bases. The FLIM image reconstruction and lifetime analysis was performed by following the previous method^47^ using custom written software (TTTR Data Analysis, developed in LabVIEW, NI). When a high spatial resolution was not required (for example for Figs 1c–f and 2c–e), a 32 pixels × 32 pixels format was used for FLIM acquisition. For FLIM imaging with high spatial resolution (for example for Figs 2b and 4a,b), a 512 pixels × 512 pixels image format was employed. A minimum of 200 photons per pixel was acquired for all FLIM acquisitions. FLIM images were generated by plotting the average photon arrival time for each pixel. The instrument response function (IRF) was determined by measuring the reflected flight from a Ludox solution (Sigma Aldrich). The notch filter for the excitation laser line and the emission band pass filter were removed from the microscope for collection of the reflected light.

To fit lifetime data, photons from the entire image were grouped into a single decay curve so that each decay curve contained >200,000 photons. This was done to increase the robustness of the fit. We started by tail fitting the decay curves between 1.2 and 20 ns as indicated by the dotted box in Supplementary Fig. 1a, first to a single component exponential decay function, then a double-component exponential decay function and finally a triple-component exponential decay function. As illustrated by the residuals in Supplementary Fig. 1b, even a double-component exponential decay function was insufficient to capture the fluorescence lifetime decay of Lck10-CliF at high expression levels or under conditions when CY2PHR-CliF and CD3ζ-CliF were clustered (data not shown). In contrast, a triple-component exponential decay function with a short lifetime of 0.5–0.6 ns, an intermediate lifetime of 1.5–1.9 ns and a long lifetime of ∼3 ns resulted in similar high quality fits for all experimental conditions.

Data were tail-fitted to a triple exponential decay function of


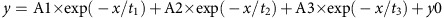


between the lifetime of 1.2 to 20 ns using the Levenberg Marquardt method in Originpro software. This fitting approach avoided the influence from IRF, which did not extend beyond 1.2 ns. After the lifetime value of best fit was retrieved, the lifetime values were fixed and the relative fractions of individual lifetimes were plotted. The fraction was calculated from the amplitude of the given lifetime as a percentage of the total amplitudes of all lifetimes. For comparisons, all FLIM data were forced fitted to a triple exponential decay function with lifetimes fixed at the indicated values. For CliF, fixing two lifetimes and leave the third one free produced similar results (Supplementary Figs 2,5 and 8). For the donor-only data (with and without co-expression of the acceptor in the same cell) shown in Figs 1e,f and 2e, we also fitted lifetimes to a double-exponential decay function; these results are shown Supplementary Figs 2 and 5. The average donor lifetime was acquired by taking the average photon arrival time of the entire TCSPC intensity histogram.

We also verified that that the tail fitting approach was suitable, by comparing it to an iterative reconvolution fitting approach. The latter considers the lifetime decay as a convolution of the experimentally determined IRF and multiple exponential decay functions. For our data, the reconvolution fit could describe the decay from 0.7 to 20 ns but again required a triple-component exponential decay function to obtain a reasonable fit. The χ^2^ values for tail fitting and iterative convolution taking the IRF into account were comparable and we thus used tail fitting throughout.

The reconvolution fit was performed using FluoFit (PicoQuant) following the procedure described by Enderlein and Erdmann^48^. This method simulates the decay by a convolution of the experimental IRF, *IRF(t)* with a user-defined exponential decay model *I*_model_ *(t)*, as 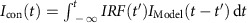. Fitting was performed by minimizing the difference between the simulated data and the experimental data using a linear least square minimization method.

To compare the lifetime values of the non-clustered and clustered TCR in Fig. 4d, two mask FLIM files were created by thresholding the original FLIM image by photon intensity. The average lifetime values of the two mask files were compared by paired *t*-test.

To retrieve the cluster density of TCR clusters described in Supplementary Fig. 10, the FLIM image of CD3ζ-CliF was converted to a FRET efficiency image according to 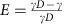. Three non-clustered regions were randomly selected from the FLIM image, and the FLIM image was recalibrated so that the lifetime values of those regions were forced to 2.75 ns as the lifetime of monomeric CliF. The cluster density was directly retrieved from the FRET efficiency values of the calibrated FRET image using equation (1) described in the Supplementary note.

### Data availability

Data and MATLAB codes are available upon request to the corresponding author.

## Additional information

**How to cite this article:** Ma, Y. *et al*. An intermolecular FRET sensor detects the dynamics of T cell receptor clustering. *Nat. Commun.* **8,** 15100 doi: 10.1038/ncomms15100 (2017).

**Publisher's note**: Springer Nature remains neutral with regard to jurisdictional claims in published maps and institutional affiliations.

## Supplementary Material

Supplementary Information Supplementary Figures, Supplementary Table, Supplementary Note and Supplementary References

Supplementary Movie Light induced clustering of CY2PHR-CliF in transfected COS-7 cells. The movie is produced from the merged image series of donor (green) acceptor (red) by 488 nm light irradiation at frame rate of 1 frame/s.
