# Supplementary material for: An intermolecular FRET sensor detects the dynamics of T cell receptor clustering
Source: Nat Commun. 2017 Apr 28;8:15100. doi: 10.1038/ncomms15100 (PMC5414349; doi:10.1038/ncomms15100)
Supplement: Supplementary Information — Supplementary Figures, Supplementary Table, Supplementary Note and Supplementary References [file ncomms15100-s1.pdf]

Supplementary Note

Calcium-induced CD3ζ-CliF clustering

We sought an experimental condition under which TCR-CD3 clustering could be controlled externally and independently from receptor stimulation without having to artificially crosslink or cluster proteins. Surprisingly, we found that calcium influx induced by ionomycin treatment was sufficient to induce CD3ζ-CliF clustering (**Supplementary Fig. 9a**). As intracellular calcium fluxes can induce other effects such as actin reorganization, we prepared membrane lawns by adhering CD3ζ-CliF expression COS-7 cells onto coverslips and ripping off the cell body. This leaves an intact membrane sheet on the coverslip in which proteins are still laterally mobile[1]. Exposure of the membrane lawns to high concentration (2 mM) of calcium triggered the formation of CD3ζ-CliF clusters[2] (**Supplementary Fig. 10a**). Within 15 sec, FRET efficiency (excitation and emission crosstalk-corrected R/G ratio[3]) of CliF rapidly gained from 0.16 to 0.45, and reaching a stationary phase at its maximum afterwards (**Supplementary Fig. 10b**). Smaller CD3ζ-CliF clusters could also be induced by the physiological concentration of 50 µM $CaCl_2$ albeit at a slower rate (**Supplementary Fig. 9b**). This suggests that calcium-induced TCR clustering may indeed occur under physiologically relevant conditions.

Estimation of packing density of TCR-CD3 in clusters

We tried to estimated the maximum number of acceptors a CD3ζ-CliF molecule could transfer its energy to and the Förster radius at maximum FRET efficiency because these parameters can inform on the highest packing density of CD3ζ-CliF within TCR-CD3 clusters. Both theoretical and experimental studies of homo-FRET and hetero-FRET showed that the overall FRET efficiency depends on the number of acceptors surrounding excited donors within the cluster[4, 5, 6, 7, 8, 9]. For CliF, the excited donor can transfer energy to both its own acceptor and neighbouring acceptors. In the calculations below, we made the assumptions that energy transfer to its own acceptor *via* intramolecular FRET does not change with clustering and that the intermolecular transfer rate depends on the number of other CliF molecules within the cluster. We also made the assumption that only FRET and no other environmental factors influenced fluorescence lifetime values. The transfer efficiency of a cluster can then be described as

$$E_{cluster} = \frac{k_{intra} + n k_{inter}}{k_{intra} + n k_{inter} + \frac{1}{\gamma D}} \qquad \text{(Equation 1)}$$

where k_{intra} is the intramolecular transfer rate, k_{inter} the intermolecular transfer rate, n the average number of molecules to which an excited donor can transfer energy and γ^D is the donor lifetime in absence of acceptor. It is further assumed that each energy transfer occurs independently and that the distances between FRET pairs within the cluster are constant. Therefore, when the maximum number of acceptors occupies the area within the Förster radius of a central donor molecule, the addition of further molecules does not increase the FRET efficiency. Therefore, $E_{cluster}$ becomes E_{max} for $n > n_{max}$ with both E_{max} and n_{max} being constant. By fitting this equation to the experimental data (**Supplementary Fig. 10b**), we obtained values for $k_{intra} = 0.036$, $k_{inter} = 0.0198$ and $n_{max} = 11$. Based on the assumptions made, this means that there were 11 acceptor molecules within the Förster radius of each donor molecules inside the CD3ζ-CliF clusters. A previous study[7] suggested that the Förster radius R_n of multiple acceptors is given by

$$R_n = (n^{1/6} * R_0) \qquad \text{(Equation 2).}$$

The normal Förster radius R_0 between single Venus and single mCherry is 5.66 nm[10]. That would mean that in CD3ζ-CliF with 11 acceptors per donor molecules, the Förster radius R_n = 8.4 nm for n = 11. Given that $k_{inter}/(1/\gamma^D) = (R_n/R)^6$, we derived the minimal molecular distance between CD3ζ-CliF

molecules as 13.45 nm. This equates to a molecular footprint of 142 nm^2 and a radius of 6.73 nm. Given that the footprint of the transmembrane domains of TCR-CD3 complex was reported to be ~45-55 Å[11] and 65 Å[12], it is highly likely that at this density the cytosolic tails of CD3ζ and CD3ε are not attached to the plasma membrane so that this density reflects a signalling competent state of the TCR-CD3 complex.

We also estimated the relative density n of each cluster within an image (i.e. using the values for k_{intra} and k_{inter} values established above in Equation 1). To do so, we measured FRET efficiency with donor FLIM imaging due to its high sensitivity. Given that

$$E = \frac{\gamma D - \gamma}{\gamma D} \qquad \text{Equation (3)}$$

where γ is the donor lifetime in presence of acceptors, a calibrated FRET efficiency map was produced from the FLIM image (**Supplementary Fig. 10c, d**, see Methods). Thus, the cluster density (i.e. the average number of acceptor molecules within the Förster radius R_n = 8.4 nm) could be directly extracted from the FRET efficiency map as indicated by the highlighted clusters in **Supplementary Fig. 10c, d**. When CD3ζ-CliF clusters in resting and activated conditions were compared, we found that the cluster density was higher under activating condition (**Supplementary Fig. 10e, f**). In resting T cells, CD3ζ-CliF had an average density of 7.6 ± 1.8 molecules per Förster radius, while in activated T cells, clusters had 9.0 ± 2.3 molecules per Förster radius (n=100 clusters, P<0.01). It was particularly noticeable that only activated T cells had CD3ζ-CliF clusters with $n_{max} \geq 11$ that were also observed in Ca^{2+}-induced TCR clustering in the membrane lawns. However, these numbers however should be regarded with caution, given the assumptions made.

Supplementary Figures

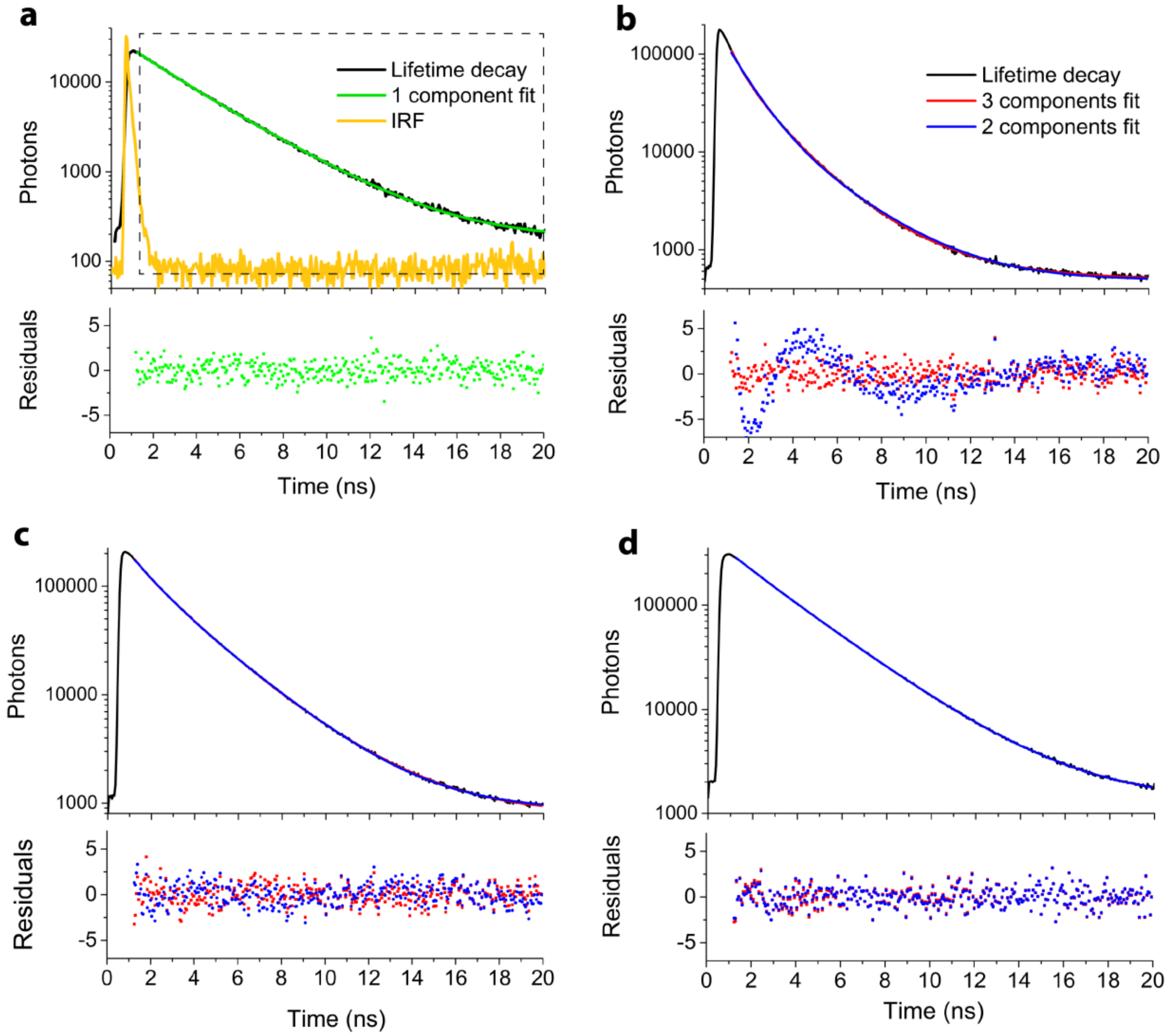

Supplementary Figure 1. Lifetime decays of Venus in Lck10-Venus (a, d), Lck10-CliF (b) and co-expressed Lck10-Venus and mCherry-H-Ras11 (c) fitted single exponential decay (a) and double (blue) and triple (red) exponential decay (b-c) curves. Symbols are measurements, solid lines are fitting curves and residuals are shown below each decay curve. (**a**) Tail-fitting in the window of 1.2 – 20 ns (dashed box) of Venus lifetime in Lck10-Venus to a single exponential decay function. The fit produced a lifetime of 2.97 ns with $R^2 = 0.996$. The instrument response function (IRF, yellow) was measured from the reflected light of a Ludox solution. (**b**) Fitting of Venus lifetime in Lck10-CliF to a triple exponential decay function (red) produced lifetimes of 3 ns, 1.55 ns and 0.5 ns with $R^2 = 0.999$ and fitting to a double exponential decay function (blue) produced lifetimes of 2.67 ns and 1.0 ns with R^2=0.983. (**c**) Fitting Venus lifetime in Lck10-Venus co-expressed with mCherry-H-Ras11 to a triple exponential decay function (red) yielded lifetimes of 3 ns, 1.7 ns and 0.5 ns with $R^2 = 0.999$ and a double exponential decay function (blue) resulted in lifetimes of 2.69 ns and 1.13 ns with $R^2 = 0.999$. (**d**) Fitting of Lck10-Venus to a triple exponential decay function (red) resulted in lifetimes of 3 ns, 1.9 ns and 0.5 ns with $R^2 = 0.999$ and fitting to a double exponential decay function (blue) yielded lifetimes of 2.98 ns and 1.76 ns with $R^2 = 0.999$. The residuals indicate that Lck10-CliF (b) ought to be fitted to a triple exponential decay function. Data are representative of at least 20 cells for each condition.

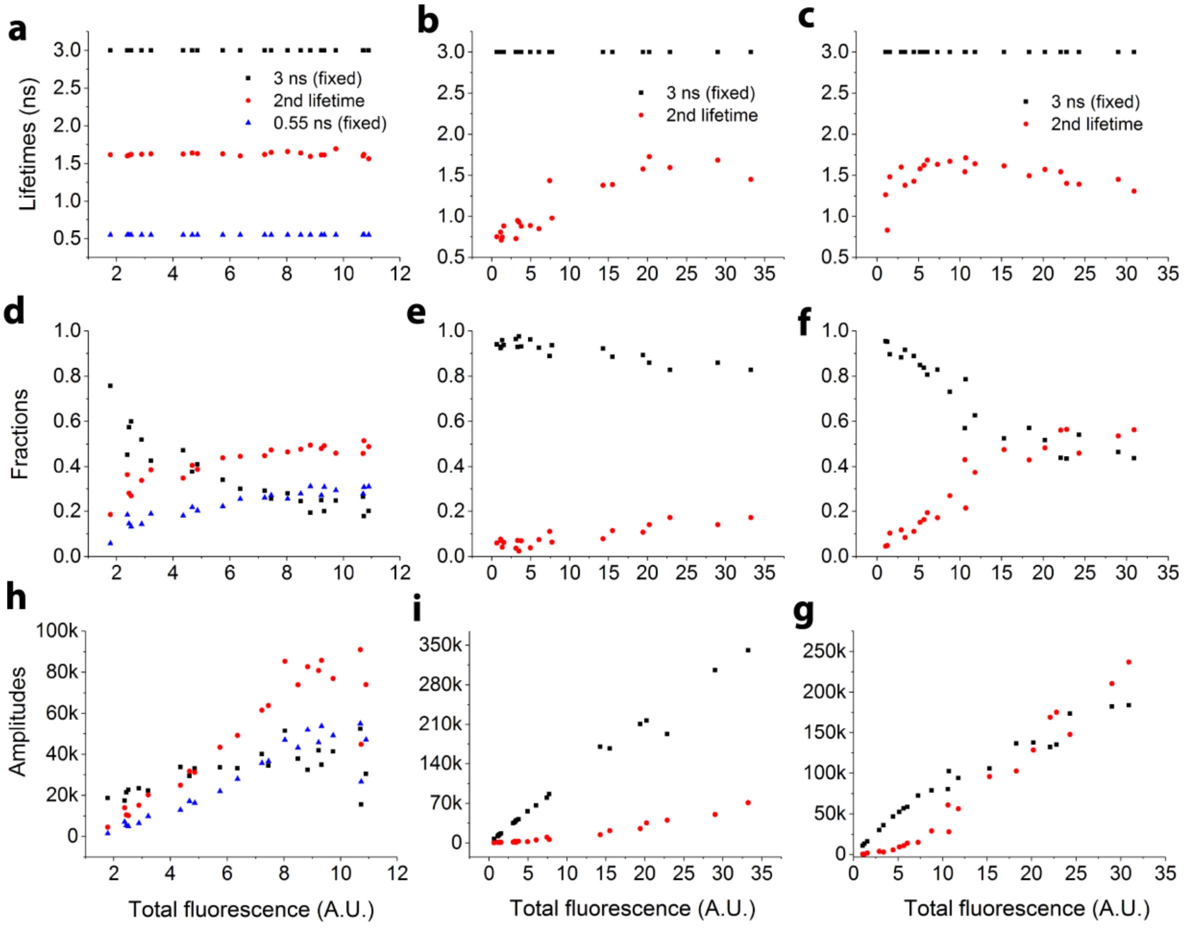

Supplementary Figure 2. Alternative fitting procedures applied to data shown in Figure 1 d, e f. (**a-g**) Venus lifetimes (**a-c**) and corresponding relative fractions (**d-f**) and absolute amplitudes (**h-g**) as a function of total (donor + acceptor) intensity for Lck10-CliF (**a, d, h**), Lck10-Venus (**b, e, i**), and co-expressed Lck10-Venus and mCherry-H-Ras11 (**c, f, g**). Lifetime values for Lck10-CliF (**a**) were acquired by fitting to three-component exponential decay functions where two lifetimes at 3 ns and 0.55 ns were fixed, and the third left free. The free, intermediate lifetime of Lck10-CliF was 1.62 ± 0.03 ns across the intensity range. Please note that the absolute amplitude of the 3 ns lifetime in Lck10-CliF (black symbols in **h**) changed relatively little as a function of expression level, in contrast to the amplitudes corresponding to the shorter lifetimes (red and blue symbols), suggesting that the 3 ns lifetime may represent non-FRETing sensors with an non-functional or poorly aligned acceptor. Lifetimes for Lck10-Venus (**b, e, i**), and co-expressed Lck10-Venus and mCherry-H-Ras11 (**c, f, g**) were fitted to a two-component exponential decay function with one lifetime at 3 ns fixed and the other left free. Color legend shown in (**a-c**) also applies to (**d-g**). Data were from at least 20 cells per condition.

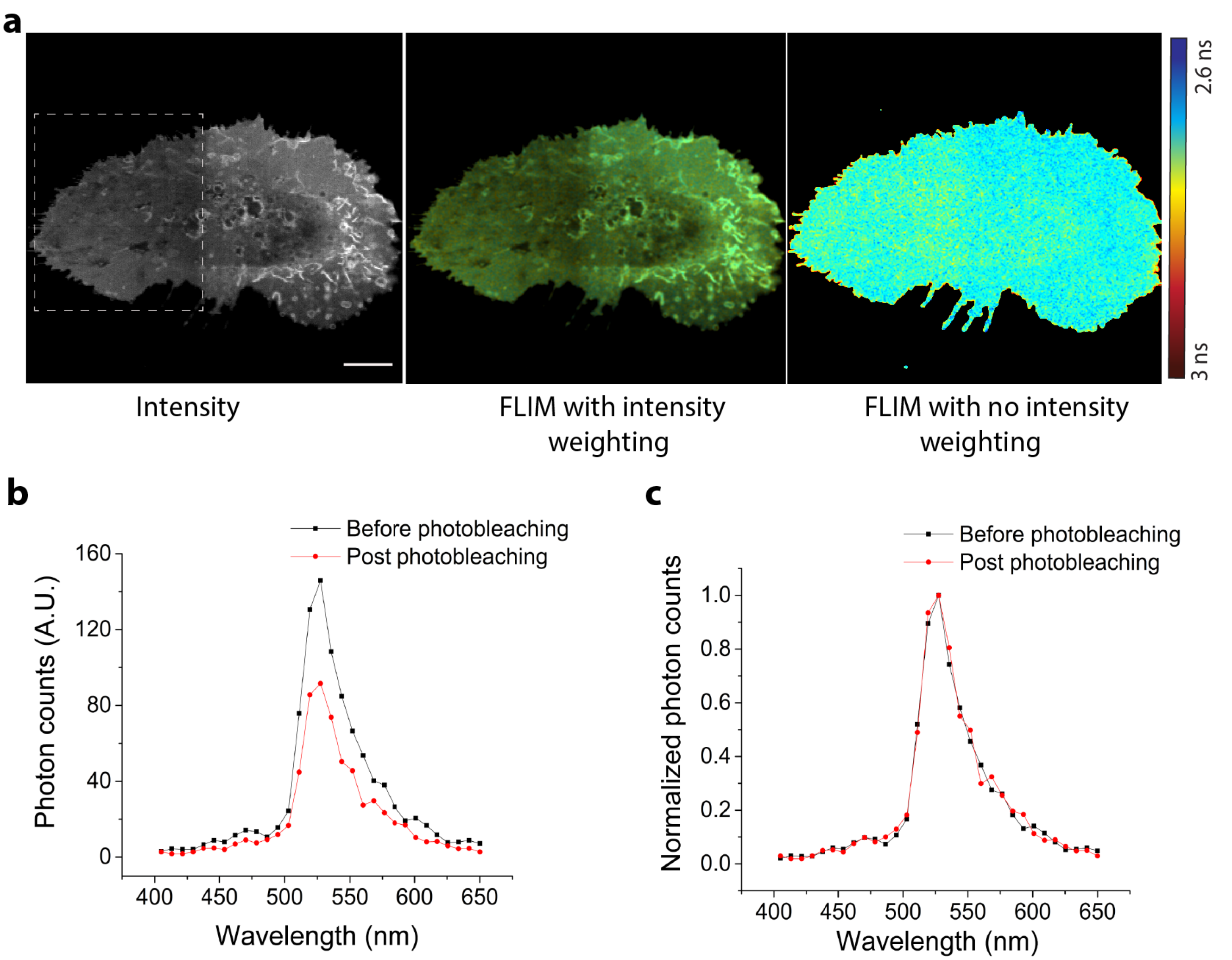

Supplementary Figure 3. Photobleaching of Venus did not change its lifetime or fluorescence spectrum. (**a**) Intensity and photon-weighted and non-weighted FLIM images of a fixed COS-7 cell expressing Lck10-Venus. The dotted line indicates the region that was photobleached with 488 nm light at 60% above the initial intensity. Both the photon-weighted and non-weighted FLIM images did not display a change in Venus lifetime. Scale bar = 3 µm. FLIM images were colour coded from blue to red as indicated by the colour scale. (**b, c**) Emission spectrum of Lck10-Venus in COS-7 cells before and after photobleaching. The entire cell was photobleached with 488 nm light (60% above initial intensity). Venus was excited with 2-photon excitation at 800 nm and emission spectra collected over the range of 400-650 nm before (black symbols and curve) and after photobleaching (red symbols and curve). The normalized emission spectra in **c** indicate that there was no light-induced photoconversion of Venus.

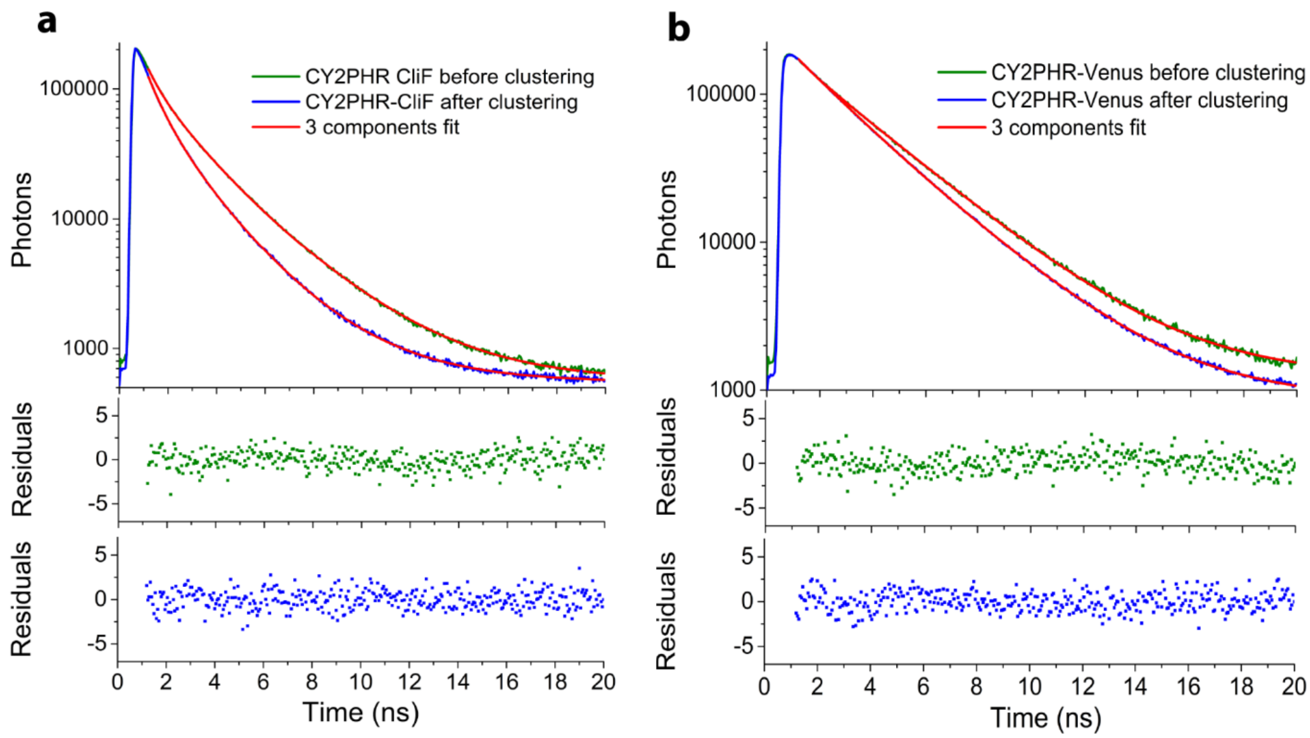

Supplementary Figure 4. **Fitting of Venus lifetime decays in CY2PHR-CliF and CY2PHR-Venus before and after irradiation with blue light to induce protein clustering.** (**a, b**) Donor lifetime decay of CY2PHR-CliF (**a**) and CY2PHR-Venus (**b**) before (green line) and after (blue line) light-induced protein clustering of CY2PHR and fitting of decays to 3 component exponential decay function (red line). Best fits result in lifetime values of 3 ns, 1.55 ns and 0.5 ns (before clustering), and 3 ns, 1.55 ns and 0.6 ns (after clustering) for CY2PHR-CliF, and 3 ns, 1.9 ns and 0.5 ns for CY2PHR-Venus both before and after clustering. R^2 = 0.999 for all fits. Data are representative of 20 cells for each condition.

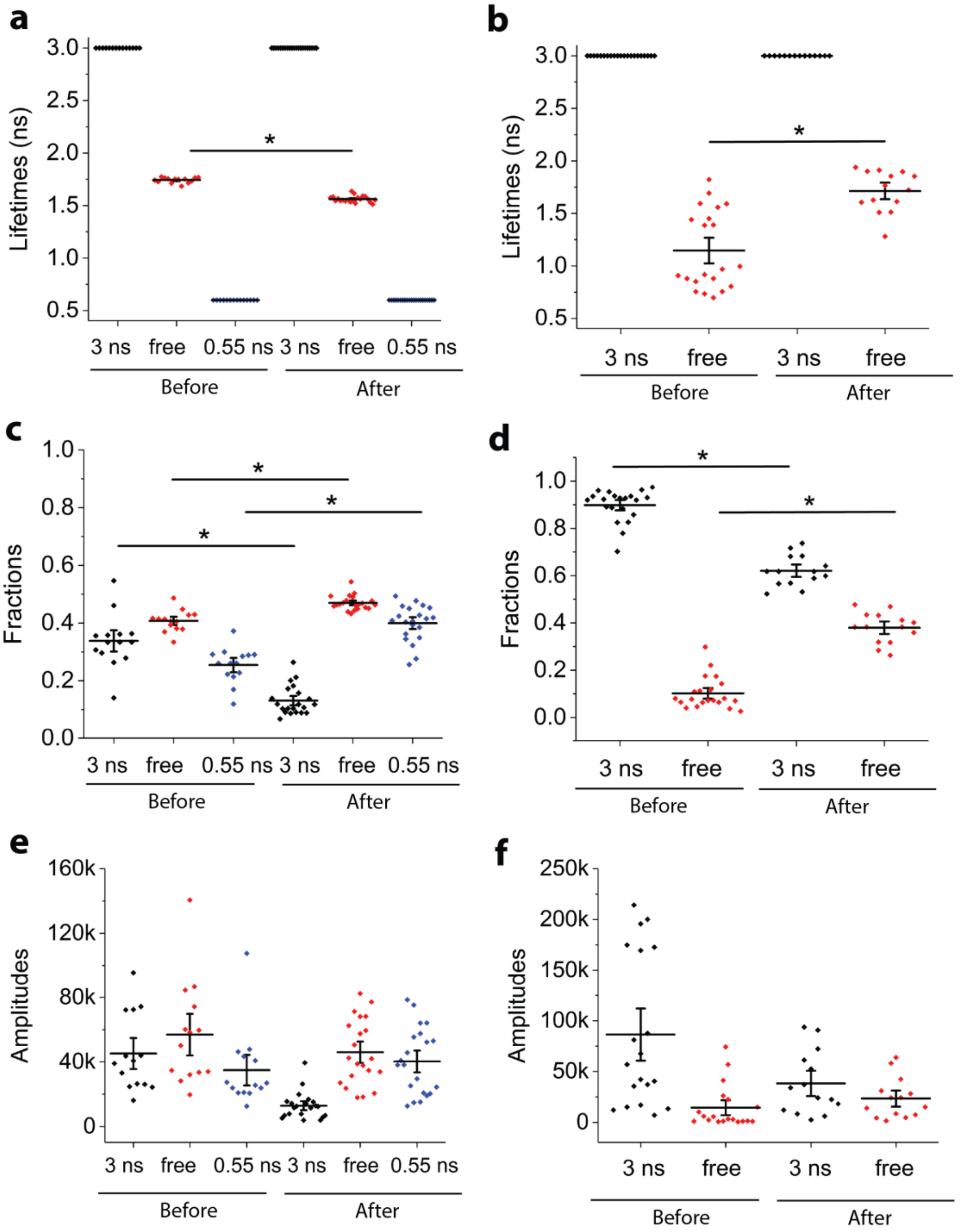

Supplementary Figure 5. Alternative fitting procedure for data in Figure 2 d-e. (**a-f**) Venus lifetime (**a-b**), corresponding relative fractions (**c-d**) and absolute amplitudes (**e-f**) in CY2PHR-CliF (**a, c, e**) and CY2PHR-Venus (**b, d, f**) before and after light-induced clustering. Lifetimes of CY2PHR-CliF were fitted to a three-component exponential decay function where two lifetimes (3 ns and 0.55 ns) were fixed and the third left free. Lifetimes for CY2PHR-Venus were fitted to a two-component exponential decay function with one lifetime (3 ns) fixed and the other left free. The free lifetime of CY2PHR-CliF before and after clustering were 1.74 ± 0.25 ns, 1.56 ± 0.29 ns and for and CY2PHR-Venus 1.15± 0.37 ns and 1.71 ± 0.20 ns, respectively. Horizontal bars indicate means and error bars are SEM. Data are from at least 15 cells per condition; *P < 0.01 (unpaired t-test).

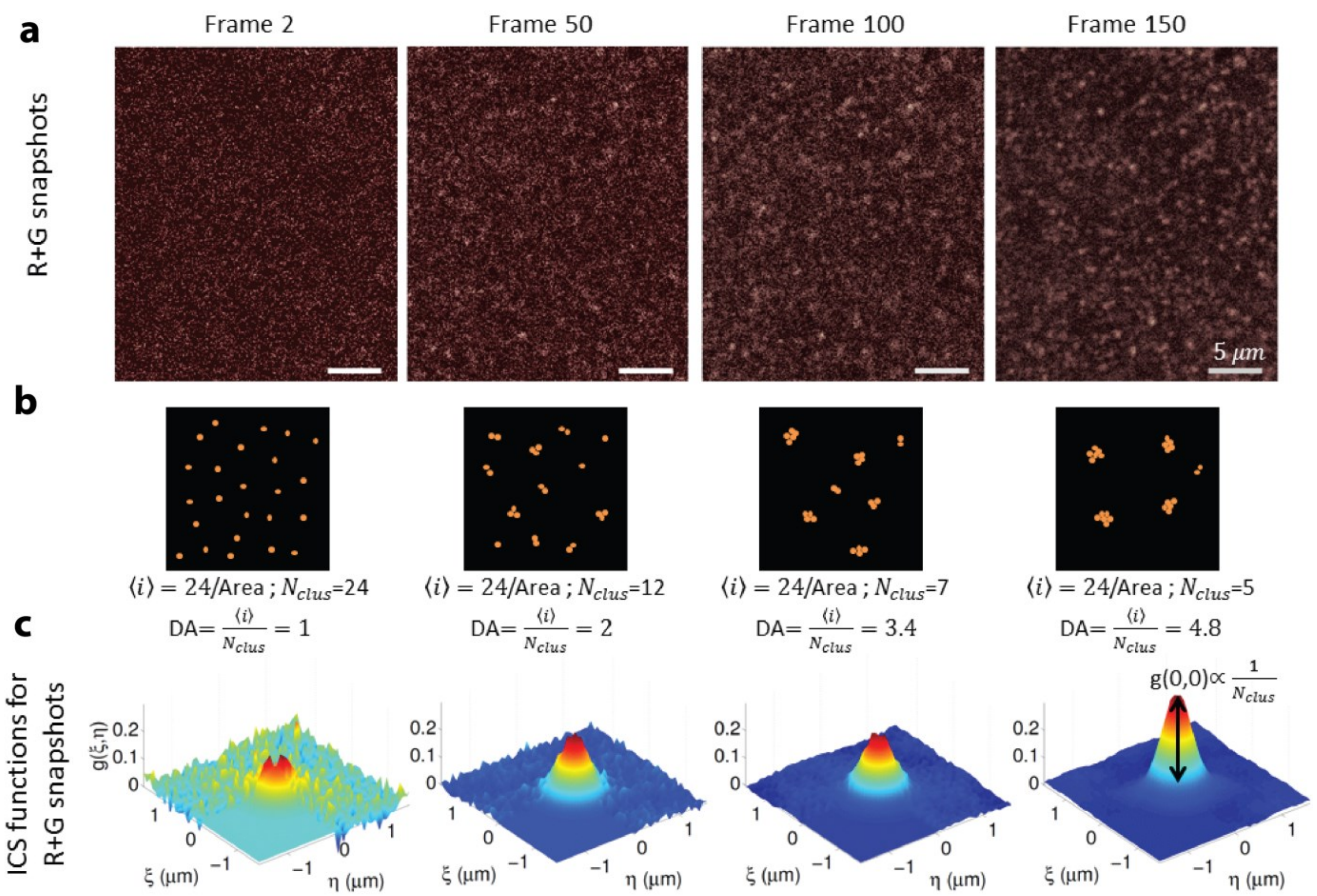

Supplementary Figure 6. Image correlation spectroscopy (ICS) analysis of time series of CY2PHR-CliF undergoing clustering. (a) Snapshots of light-induced CY2PHR-CliF clustering at indicated frame numbers. The increased scale of protein clustering can be directly visualized. (**b**) A schematic simulation of spatial aggregation of 24 molecules in the defined area over time. <*i*> and N_{clus} stand for number of molecules and number of clusters in the defined area. DA stands for Degree of Aggregation, which is the ratio between <*i*> and N_{clus}. As molecules become clustered, <*i*> stays constant, N_{clus} is reduced, and DA is increased. (**c**) Spatial image auto-correlation function (ACF) analysis of CY2PHR-CliF images at the corresponding frames in (a). The significance of the ACF function is increased due to the decrease of features that cause fluctuations. The amplitude of ACF is inversely proportional to the number of features (clusters) in the image.

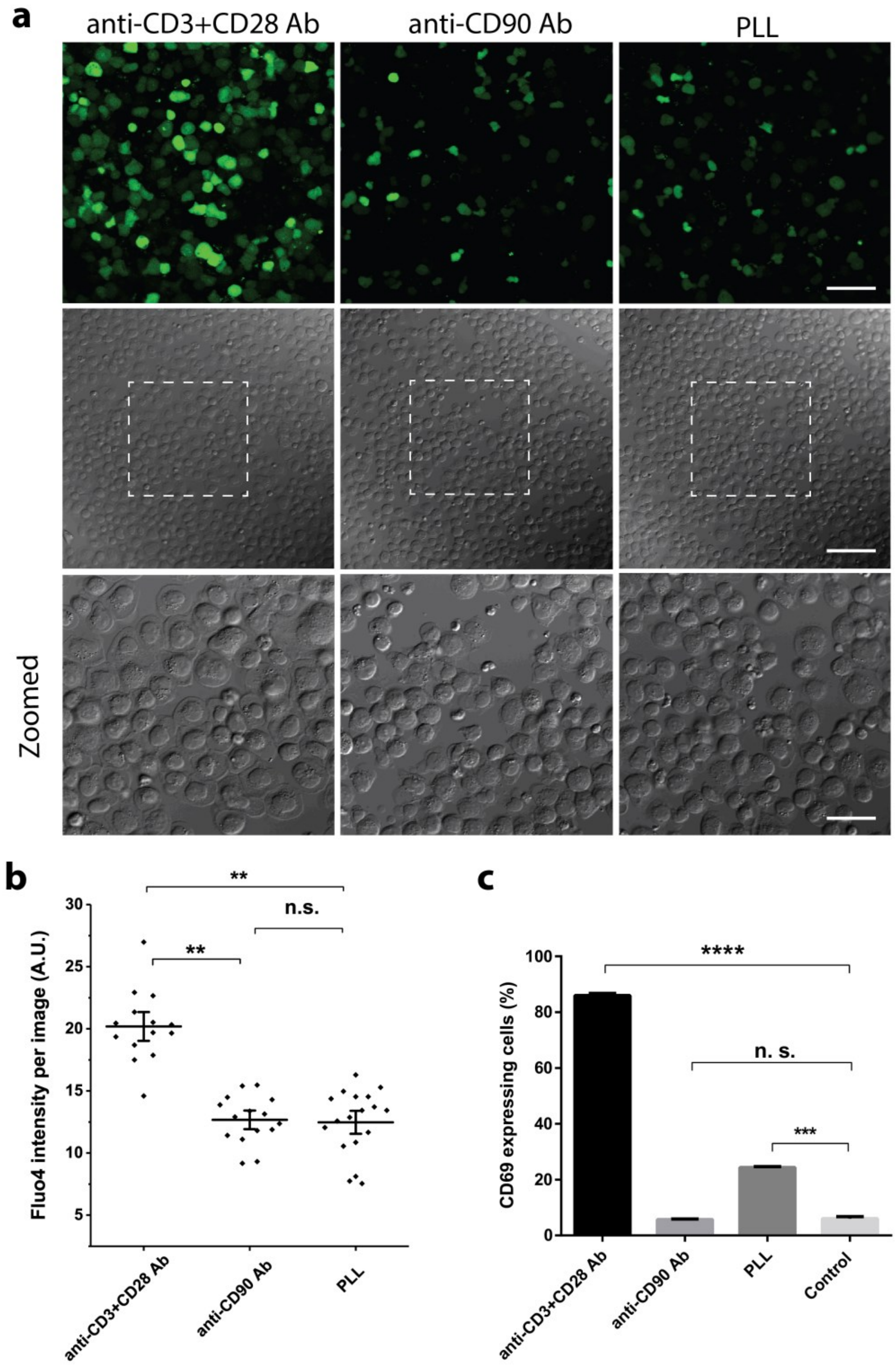

Supplementary Figure 7. Anti-CD90 Ab coated surfaces did not activate Jurkat T cells. Cells were exposed to surfaces coated with anti-CD3 and anti-CD28 antibodies (anti-CD3+CD28 Ab), anti-CD90 antibodies (anti-CD90 Ab) or poly-L-lysine (PLL). (**a**) Confocal and differential interference contrast (DIC) images of Jurkat cells loaded with Fluo4 on the indicated surfaces after 15 minutes of incubation. Jurkat cells exhibited spontaneous calcium fluxes even on non-activating surfaces, however the number of cells with elevated intracellular calcium levels was higher on activated anti-CD3+CD28 Ab surfaces. Zoomed DIC images (third row) indicated cell spreading. Scale bar (top and middle row)

= 80 µm; scale bar (bottom row) = 40 µm. (**b**) Average Fluo4 fluorescence intensity per image for T cells on the indicated surfaces (n = 12 per surface). Horizontal bars indicate means and error bars are SEM. There were ~300 cells per image. (**c**) Percentage of cells expressing CD69 after incubation in suspension (control) or on the indicated surfaces for 12 h. Cells were stained with anti-CD69 antibody conjugated to Alexa488 and analysed by flow cytometry. Bar indicate means and error bars are SEM. One way ANOVA with Bonferroni post hoc test was used for the statistical analysis in b and c; n.s., not significant when $P > 0.05$; ** $P < 0.01$; *** $P < 0.001$ and **** $P < 0.0001$.

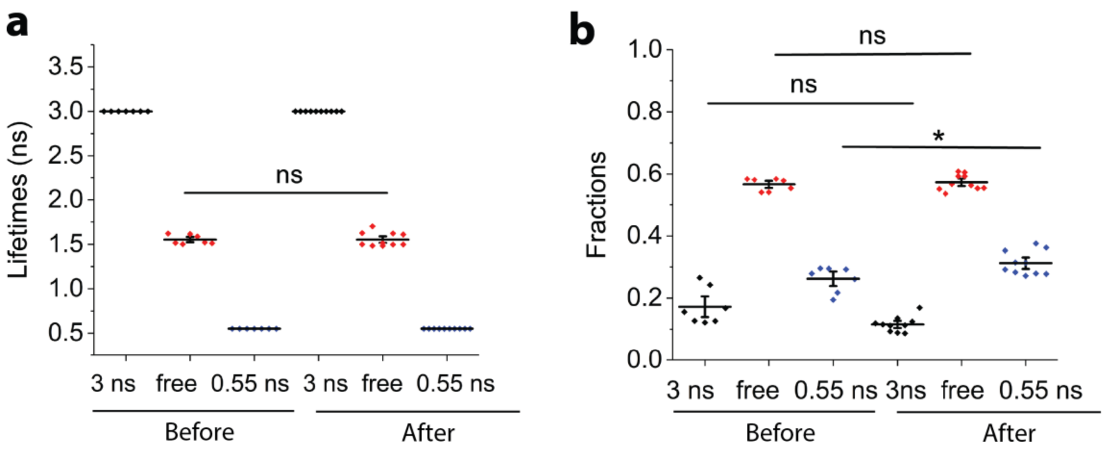

Supplementary Figure 8. Alternative fitting procedure for data shown in Figure 4 c. (**a-b**) Venus lifetime (**a**) and corresponding fractions (**b**) of CD3ζ-CliF in resting (glass surface coated with anti-CD90 antibodies) and activating (glass surface coated with a mixture of anti-CD3 and anti-CD28 antibodies) conditions. Lifetime values of CD3ζ-CliF were acquired by fitting to a three-component exponential decay function where two lifetimes (3 ns and 0.55 ns) were fixed and the third left free. The free lifetime of CD3ζ-CliF in resting and activated condition was 1.55 ± 0.52 ns and 1.56 ± 0.79 ns, respectively. Horizontal bars indicate means and error bars are SEM. Data are from 13 cells per condition. ns indicate no statistical difference; $P < 0.01$.

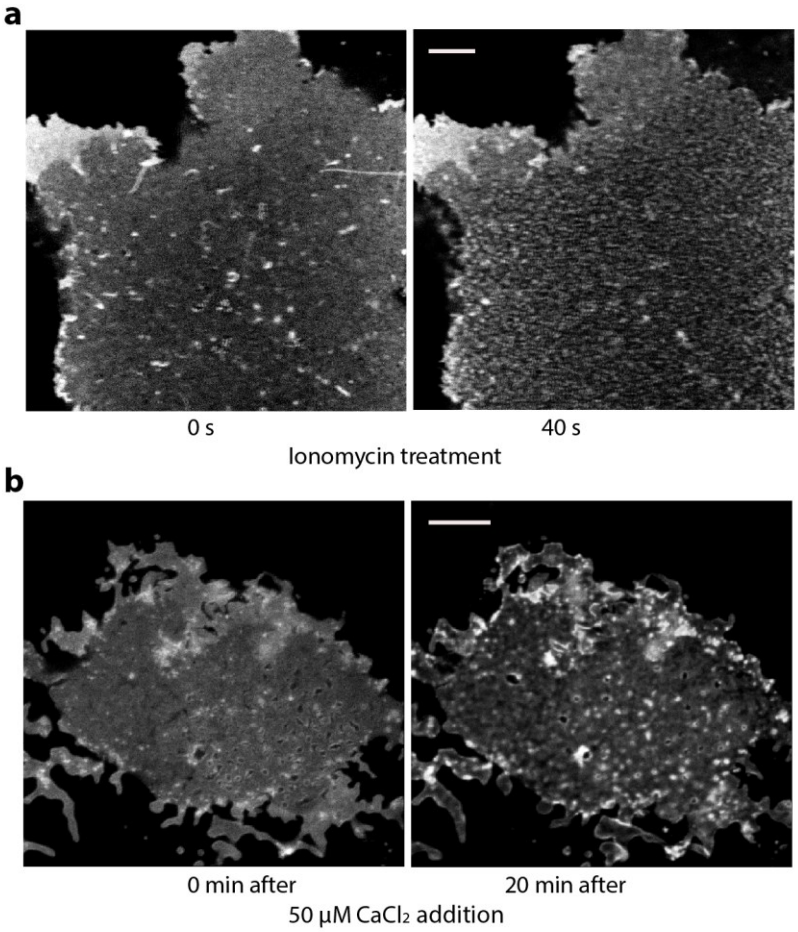

Supplementary Figure 9. Clustering of CD3ζ-CliF by Ca^{2+} influx in a live cell (a) and by $CaCl_2$ in a membrane lawn (b). (**a**) Clustering of CD3ζ-CliF in transfected COS-7 cells. Cells expressing CD3ζ-CliF were imaged in HBSS buffer that contained 1.2 mM Ca^{2+}. Ionomycin was added at a final concentration of 2 μM. Intensity images were taken at 2 s intervals, indicating the rapid spatial clustering of CD3ζ-CliF by Ca^{2+} influx. Scale bar = 2 μm. (**b**) Induction of CD3ζ-CliF clustering by $CaCl_2$ in membrane lawn. The membrane lawn was submerged in 30 mM Ca^{2+}, Mg^{2+} free PBS. 50 μM of $CaCl_2$ (final concentration) was added to the media, which induced clustering of CD3ζ-CliF after 20 minutes. Data are representative images of 5 experiments.

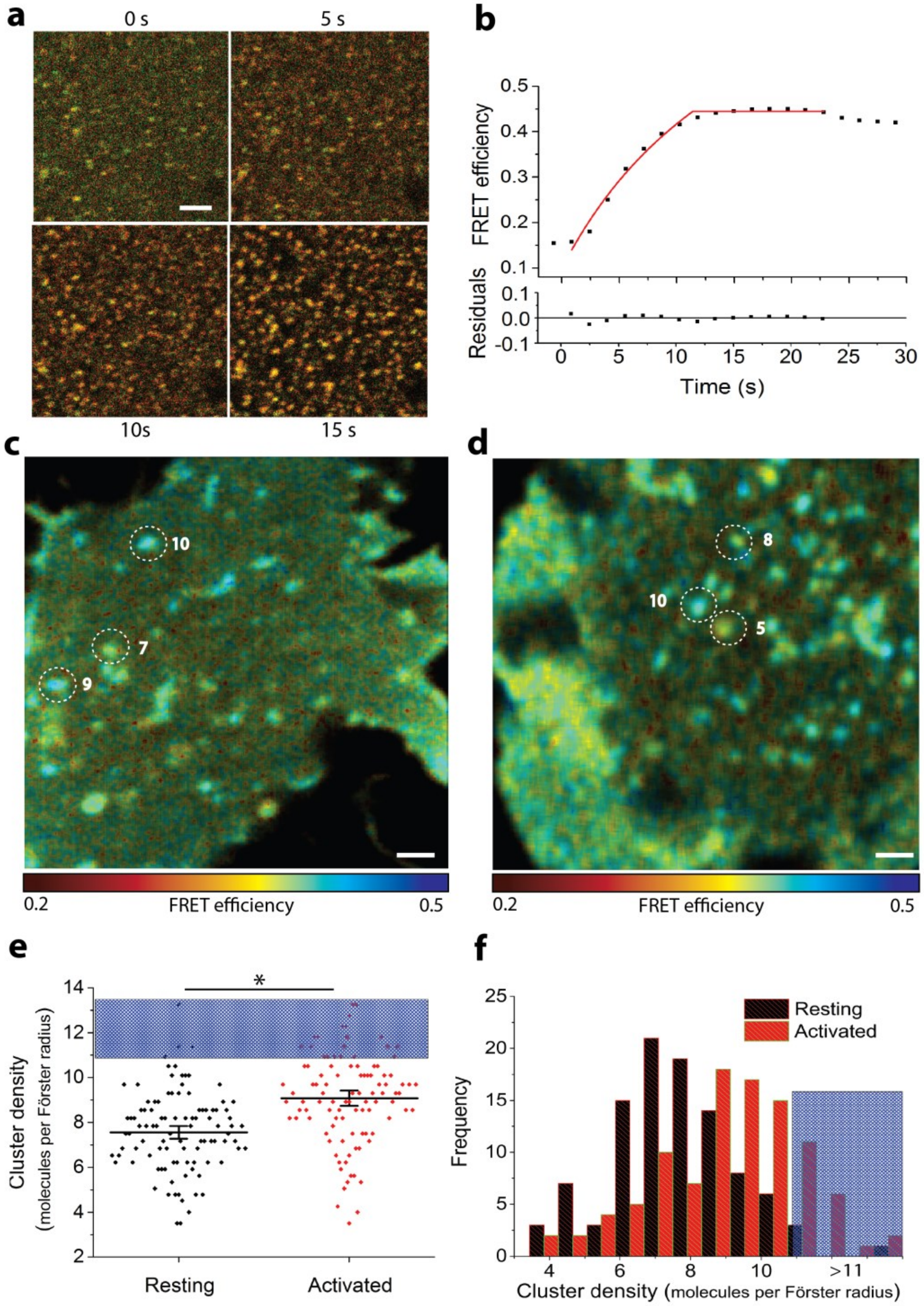

Supplementary Figure 10. Estimation of TCR cluster density. (**a**) $CaCl_2$ (2 mM)-induced clustering of CD3ζ-CliF in a membrane lawn at indicated time points (**a**). Images are merged images of donor intensity (green) and acceptor intensity (red). Scale bar is 1µm. (**b**) FRET efficiency was measured as crosstalk corrected donor/acceptor fluorescence (R/G) ratio (see Methods). Data (black symbols) were fitted to Equation 1 (red line, R^2= 0.987). Residuals are shown below. Data is representative of 3 independent experiments. (**c, d**) Estimation of TCR cluster density in resting T cells (on anti-CD90 Ab-coated surfaces, **c**) and activated T cells (on anti-CD3+CD28 Ab-coated surfaces, **d**). FRET efficiency was directly calculated from average donor lifetime values, and the average number of CliF molecules per Förster radius (indicated for selected clusters) was retrieved from Equation 1 and 3. Images in **c** and **d** are representative of 7 images collected. Scale bar is 1 µm. (**e, f**) Cluster density (**e**) and distribution

(**f**) of CD3ζ-CliF clusters extracted from the FLIM image in resting and activated conditions. In (**e**), horizontal bars indicate means and error bars are SEM. The maximum cluster density is indicated by the shaded area. Data are 100 individual clusters selected from 3 cells in each condition. *P<0.05 (Two-sample two-tailed T test).

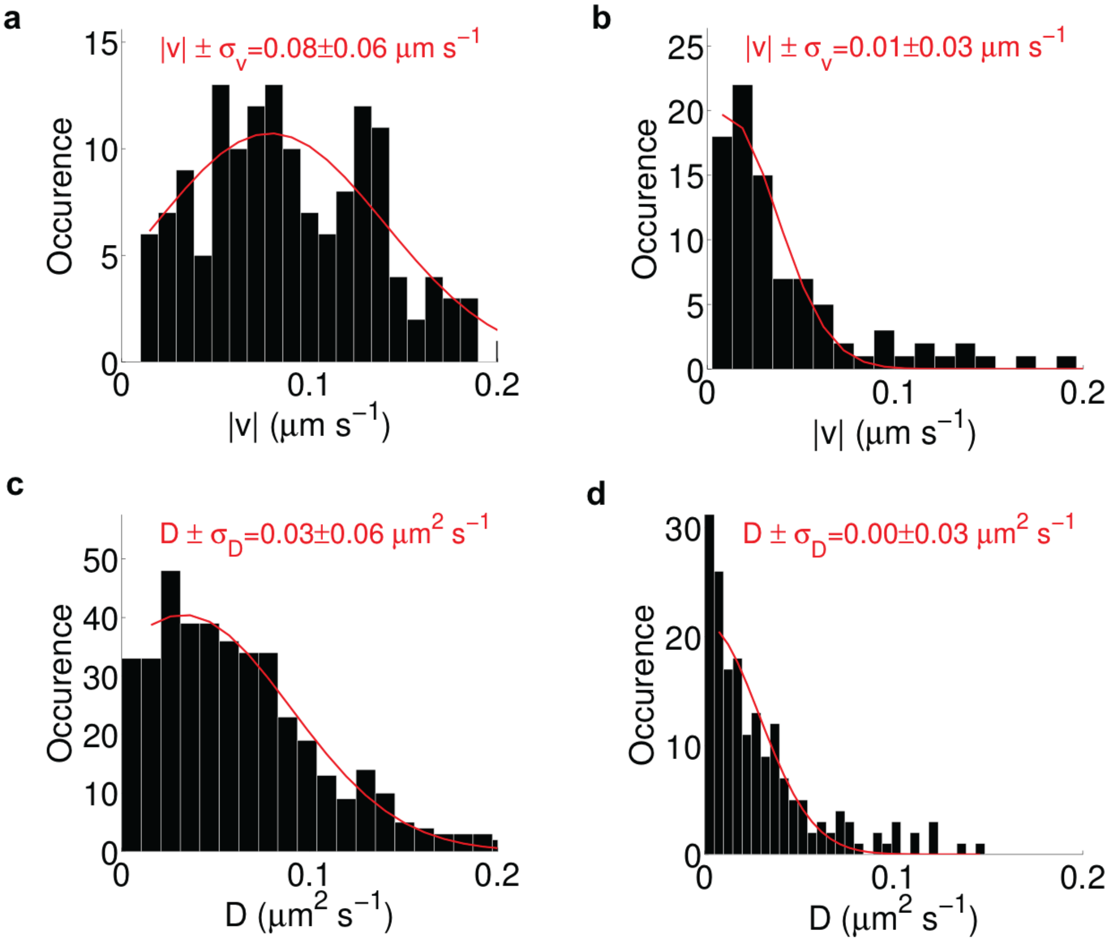

Supplementary Figure 11. Analysis of flow (a-b) and diffusion constants (c-d) of CD3ζ-CliF clusters in T cells stimulated with pMHC-presenting lipid bilayers (a,c) and antibody (anti-CD3+ CD28 Ab)-coated surfaces (b,d). The V and D values were acquired by fitting the MSD of each trajectory to a quadratic equation, see methods. The values were grouped to a histogram and the mean and standard error were extracted by fitting the histogram profile to a Gaussian function as indicated by red lines in the graph.

Supplementary Table 1 List of DNA sequences.

**Name**	**Sequence (5' to 3')**
LCK10_Venus_EcoRI FW	ATT GAA TTC ATG GGC TGT GTC TGC AGC TCA AAC CCT GAA GGA GGA GGA GGG GAT CCT ATG GTG AGC AAG GGC GAG GAG CTG TT
Venus ΔC11 BspEI RV	AAT TCC GGA GGC GGC GGT CAC GAA CTC CAG CA
Cherry_N7 BspEI FW	TAT TCC GGA GAT AAC ATG GCC ATC ATC AAG GAG TTC ATG CGC TT
mCherry NotI RV	GTC GCG GCC GCT TAC TTG TAC AGC TCG TCC ATG CCG CCG GTG GAG TG
Venus C11 2A RV	CGG CTT GTT TCA GCA GGC TAA AGT TAG TAG CTC CGC TTC CGG CGG CGG TCA CGA ACT CCA GCA G
2A cherry N7 FW	AGC CTG CTG AAA CAA GCC GGA GAC GTG GAG GAG AAC CCT GGA CCT GAT AAC ATG GCC ATC ATC AAG GAG TTC AT
Venus_Cy2_OVLP RV	GCC CTT GTA CAG CTC GTC CAT GCC GGC GGC GGT CAC GAA CTC CAG CAG
Cy2PHR OVLP Fw	GGC ATG GAC GAG CTG TAC AAG GGC TCA ACT GGA AGT ACA GGA ACA ATG AAG ATG GAC AA
Venus NotI RV	ATT GCG GCC GCT TAC TTG TAC AGC TCG TCC ATG CCG A
TCR XhoI FW	ATT CTC GAG GCC ACC ATG AAG TGG AAG GCG CTT TTC A
TCR KpnI RV	AAT GGT ACC ACT TCC ACC GCC TCC AGA ACC GCG AGG GGG CAG GGC CTG CAT GT
Lck10 OVLP RV	ATC CCC TCC TCC TCC TTC AGG GTT TGA GCT GCA GAC ACA TCC CAT GGT GGC CTC GAG ATC TGA GTC
Lck10 TCRcyto OVLP FW	AGG AGG AGG AGG GGA TAG AGT GAA GTT CAG CAG GAG CGC AGA
Cy2PHR SgrAI FW	CTC CAC CGG CGG CAT GGA CGA GCT GTA CAA GGG CTC AAC TGG AAG TAC AGG AAC AAT GAA GAT GGA CAA AAA GAC T
Cy2PHR NotI RV	GTC GCG GCC GCT CAT TTG CAA CCA TTT TTT CCC A
OTI STOP GFP FW	CCCATCCTGGTCTAGCTGGACGGCG
OTI STOP GFP RV	CGCCGTCCAGCTAGACCAGGATGGG

Supplementary References

1. Lillemeier, B.F. *et al.* TCR and Lat are expressed on separate protein islands on T cell membranes and concatenate during activation. *Nature immunology* **11**, 90-96 (2010).
2. Zilly, F.E. *et al.* Ca2+ induces clustering of membrane proteins in the plasma membrane via electrostatic interactions. *The EMBO journal* **30**, 1209-1220 (2011).
3. Chen, H., Puhl, H.L., 3rd, Koushik, S.V., Vogel, S.S. & Ikeda, S.R. Measurement of FRET efficiency and ratio of donor to acceptor concentration in living cells. *Biophys J* **91**, L39-41 (2006).
4. Runnels, L.W. & Scarlata, S.F. Theory and application of fluorescence homotransfer to melittin oligomerization. *Biophys J* **69**, 1569-1583 (1995).
5. Sienicki, K., Itagaki, H. & Mattice, W.L. On the theory of concentration depolarization of fluorescence in one- and two- component systems for multipole interactions in one, two, and three dimensional medium. *J Chem Phys* **91**, 4515-4521 (1989).
6. Li, M. *et al.* A fluorescence energy transfer method for analyzing protein oligomeric structure: application to phospholamban. *Biophys J* **76**, 2587-2599 (1999).
7. Fábián, Á.I., Rente, T., Szöllősi, J., Mátyus, L. & Jenei, A. Strength in Numbers: Effects of Acceptor Abundance on FRET Efficiency. *ChemPhysChem* **11**, 3713-3721 (2010).
8. Koushik, S.V., Blank, P.S. & Vogel, S.S. Anomalous Surplus Energy Transfer Observed with Multiple FRET Acceptors. *PLoS ONE* **4**, e8031 (2009).
9. Raicu, V. & Singh, Deo R. FRET Spectrometry: A New Tool for the Determination of Protein Quaternary Structure in Living Cells. *Biophys J* **105**, 1937-1945 (2013).
10. Akrap, N., Seidel, T. & Barisas, B.G. Förster distances for FRET between mCherry and other Visible Fluorescent Proteins. *Anal Biochem* **402**, 105-106 (2010).
11. Arechaga, I. *et al.* Structural characterization of the TCR complex by electron microscopy. *International immunology* **22**, 897-903 (2010).
12. Birnbaum, M.E. *et al.* Molecular architecture of the αβ T cell receptor–CD3 complex. *Proceedings of the National Academy of Sciences of the United States of America* **111**, 17576-17581 (2014).
